# Pursuing Diabetic Nephropathy through Aqueous Humor Proteomics Analysis

**DOI:** 10.1155/2022/5945828

**Published:** 2022-09-29

**Authors:** Huan Chen, Tan Wang, Erqian Wang, Ningning Li, Hanyi Min

**Affiliations:** ^1^Department of Ophthalmology, Peking Union Medical College Hospital, Chinese Academy of Medical Sciences and Peking Union Medical College, Beijing 100730, China; ^2^Key Laboratory of Ocular Fundus Diseases, Chinese Academy of Medical Sciences and Peking Union Medical College, Beijing 100730, China; ^3^Operating Room, Peking Union Medical College Hospital, Chinese Academy of Medical Sciences and Peking Union Medical College, Beijing 100730, China

## Abstract

In order to determine the possible aqueous humor (AH) proteins involved in diabetic nephropathy (DN) progression, we performed gel electrophoresis-liquid chromatography-tandem mass spectrometry protein profiling of AH samples from 5 patients with proliferative diabetic retinopathy (PDR) combined DN and 5 patients with PDR. Function enrichment analyses were carried out after the identification of differentially expressed proteins (DEPs). Protein-protein interaction networks were then built and the Search Tool for the Retrieval of Interacting Genes database and CytoNCA plugin in Cytoscape were utilized for module analysis. Ingenuity Pathway Analysis (IPA) was used to analyze disease and biological function, Tox function enrichment and upstream regulatory molecules/networks. Fifty-four DEPs were finally confirmed, whose enriched functions and pathways covered cell adhesion, extracellular exosome, complement activation, complement and coagulation cascades, etc. Nine hub genes were identified, including NCAM1, PLG, APOH, C3, PSAP, RBP4, CDH2, NUCB1, and GNS. IPA showed that C3 and PLG are involved in renal and urological system abnormalities. Conclusively, DEPs and hub proteins confirmed in this exploratory AH proteomic analysis may help us gain a deeper understanding of the molecular mechanisms involved in DN progression, providing novel candidate biomarkers for the early detection for diagnosis of DN.

## 1. Introduction

Diabetes mellitus is a chronic metabolic condition that can result in life-threatening complications [1–3]. Microvascular problems, like diabetic nephropathy (DN) and retinopathy (DR), are commonly associated with hyperglycemia and metabolic dysfunction in diabetes. Among microvascular complications, DN is a prime reason for end-stage renal failure globally. Currently, the clinical diagnosis of DN is based on proteinuria and/or altered glomerular filtration rate. DN progression is featured by a gradual increase in the rate of urinary albumin excretion, developing from normoalbuminuria to microalbuminuria and to macroalbuminuria. Nonetheless, due to considerable interindividual variability, conventional tests have significant limits for detection of DN in the early stages [4, 5]. Therefore, it is valuable to develop a more sensitive means to detect DN at an early stage.

Proteomics, which is based on mass spectrometry, has particular potential for identifying novel biomarkers in biofluids and could serve as the basis for new clinical testing. By analyzing the overall protein profiles in body fluids (urine, blood, etc.), proteomics can identify invaluable disease-specific biomarkers [6, 7]. In many patients with renal diseases, disease pathophysiology-related biomarkers have been identified through urine and plasma proteomic analyses, with some of them put into practical clinical application [8–10].

Because DN often follows DR in the development of microvascular complications of diabetes, risk factors for DR include diabetes control and duration, elevated blood lipid levels, race, inflammatory cytokine levels in serum, and aqueous humor (AH) [11, 12]. Thus, it is worth to set DR combined with DN as the observation group and DN as the control group to explore the AH proteins modulated by DN. However, most studies usually perform quantitative proteomic analysis on urine and plasma specimens, and it is unclear whether differences in urine protein levels across cases in these analyses are due to differences in plasma protein levels or to elevated secreted protein levels caused by kidney injury [13]. To our knowledge, no proteomic study using AH has been performed to explore the key molecules.

This research employs the protein profiling method of gel electrophoresis plus liquid chromatography-tandem mass spectrometry (GeLC-MS/MS) and conducts bioinformatic analysis of proteins with markedly changed expression among groups and aims at identifying DN-modulated AH proteins in clinically well-defined diabetic populations while highlighting the biological processes underlying disease etiopathogenesis.

## 2. Materials and Method

### 2.1. Subjects

In this prospective case series research, 10 eyes from 10 diabetic patients (5 with PDR and 5 with PDR+DN), who were examined by the same internal medicine physician between March 2019 and October 2020 in the Peking Union Medical College Hospital, were analyzed. DN was confirmed by a 24-hour urinary albumin excretion of >300 mg, and PDR was confirmed by an ophthalmologist. Patients were clinically diagnosed with active PDR, presenting with repeated vitreous bleeding and/or retinal detachment as a result of fibrovascular membrane neovascularization. This study, after obtaining the approval from the Ethics Committee of our hospital and informed consent from all participants, was conducted strictly following the Declaration of Helsinki.

Patients all underwent pretreatment ocular examinations testing intraocular pressure (IOP), axial length, best-corrected visual acuity, and corneal endothelial cell counts, as well as ultrasound biomicroscopy of anterior and posterior segments.

Cases meeting any of the following were ruled out: (1) other retinal diseases besides DR; (2) other diseases of the eyes like glaucoma; (3) intraocular inflammation or infections; (4) intraocular surgery in the past 6 months; (5) previous penetrating ocular trauma; and (6) inability to receive eye operation because of recent myocardial infarction, uncontrolled diabetes/hypertension, cerebrovascular events, etc.

### 2.2. Sample Collection and Preparation

Following informed permission, patients received three days of prophylactic topical Levofloxacin instillation. Following topical anesthetic and sterilization of the operation field, patients were given an intravitreal anti-VEGF injection (Conbercept, Aflibercept, or Ranibizumab with a dosage of 0.05 mg, 2 mg, and 0.05 mg, respectively) via the superotemporal pars plana that was located 4 mm behind the limbus. Prior to anti-VEGF therapy, AH samples were taken from each patient for 10 minutes of centrifugation (13000 g, 4°C), followed by storage in tuberculin syringes and −80°C refrigeration.

Supernatants of AH samples obtained via centrifugation were placed into three KD ultrafiltration tubes. Then the protein solution was replaced with a lysis buffer that was composed of 2 M Thiourea (Sigma-Aldrich, USA) +7 M Urea (Amresco 0568-1Kg, USA) +0.1% 3-[(3-Cholamidopropyl) dimethylammonio]-1-propanesulfonate [CHAPS] + protease inhibitors.

Following ultrafiltration and centrifugation, we collected 10 *μ*L of the sample and utilized the Bradford Protein Assay Kit (Thermo 23236, USA) for protein quantification. Proteins were then trypsin digested using the modified filter-aided sample preparation (FASP) technique [14, 15]. Briefly, lysate sample reduction was accomplished by incubating in dithiothretitol (DTT; 25 mM, Bio-Rad, USA) for 30 minutes at 60°C, and the subsequent 10 minutes of 50 mM iodoacetamide alkylation in the dark. After loading the samples onto a 10 kDa cutoff ultrafiltration membrane (Sartorius, Germany), they were incubated all night long at 37°C with trypsin (enzyme-to-protein ratio: 1 : 50). Following three 50 mM triethylammonium bicarbonate buffer (300 mL; Sigma T7408, USA) rinses, the samples were treated with 10 minutes of spinning at 12,000 g. Ziptip C18 pipette tips desalted peptides as instructed by the manufacturer's instructions.

After activation of the C18 solid phase extraction column and equilibration with ACN and 2% ACN, 0.1% FA, the sample loaded was pipetted 10 times, and then desalted and eluted with 2% and 50% ACN, 0.1% FA, respectively. After being collected into a rotary vacuum drier, the eluent was refrigerated at −80°C until use.

To build a data-independent acquisition (DIA) Spectral Library, dried peptides were subjected to 0.1% formic acid (FA; Thermo A117-50, USA) resuspension and the subsequent collection for sample dividing into samples with equal lysate quantities. The rest specimens were used with the Biognosys iRT kit, including the preparation of a 10 × iRT buffer and the subsequent addition of it to each sample at 9 : 1.

### 2.3. High-pH Reversed-Phase Fractionation

The digest samples were separated by additional high-pH reversed-phase chromatography. The RIGOL L-3000 system was utilized for the separation of mixed peptides in a 30 *μ*g digest specimen using a reverse chromatography column (RIGOL, Beijing, China). After dissolution of peptides in mobile phase A (100 *μ*L; 2% (v/v) acetonitrile (Thermo A955-4, USA), 98% (v/v) ddH_2_O, pH 10), the mixture was spun down (14,000 g) for 20 minutes.

Then the mobile phase B (98% (v/v) acetonitrile, 2% (v/v) ddH_2_O, pH 10) was injected into the supernatants at 1 mL/min in the column in a stepwise elution mode. Mobile phase B step gradients were used to acquire individual 15 minutes eluant fractions.

### 2.4. Mass Spectrometric (MS) Acquisition

For MS analysis, samples of 1 *μ*g each volume were evaluated on an EASY-nLC1000 connected to an Orbitrap Fusion™ Tribrid™ MS instrument (Thermo Scientific) with the use of an internally prepared analytical column (150 *μ*m ×150 mm, 1.9 *μ*m). A binary solvent system, which was prepared by 0.1% FA in H_2_O (A) and 0.1% FA in ACN (B), was adopted, and the linear gradient settings were as follows: 3-8% B/4 min, 8-22% B/65 min, 22-35% B/12 min, 35-90% B/4 min, and 90% B/5 min.

Using an EASY-Spray ion source, direct eluent introduction into the MS instrument was then carried out, with the spray voltage and capillary temperature set as 2.3 kV and 320°C, respectively. The whole MS scanning range was 300-1400 m/z for data-dependent acquisition- (DDA-) MS runs. With a resolution of 60,000, the MS had an under 3-stop-speed mode for 15,000 resolution MS/MS scans, while HCD had an isolation window and a normalized collision energy of 1.6 m/z and 32%, respectively. For DIA analyses, MS1 scans (automatic gain control (AGC) target 4e5 or 50 ms injection time) were performed from 300 to 1300 m/z, with DIA segmentation resolution of 30,000 (AGC target 5e5; for injection time). The collision energy was 32%, and the spectra were collected in profile mode.

### 2.5. Identification and Quantification of Proteins

DIA data analyses adopted Biognosys' Spectronaut pulsar programme [16]. The default software settings were employed for targeted data analyses, where dynamic iRT was utilized for retention time prediction types with window-based correction factors. Besides, local mass calibration, as well as limitless scrambled decoy generation, was utilized. We also employ an MS2-level interference connection for fragment elimination based on interference signals while retaining ≥3 for measurement. The false discovery rate (FDR) at peptide level was 1%.

Based on the principle of parsimony, the ID picker algorithm comes with the software package was used for proteomic inference. RAW images were converted to the Spectronaut file format when conducting spectral library-based studies and were calibrated according to the global spectral library's retention time dimension. After then, the files were used for spectrum analysis without any further retention time-based recalibration. To evaluate DDA data, Proteome Discoverer 2.3 with default settings (Trypsin/P (Promega, V5111, USA), two missed cleavages) was used. Cysteine carbamidomethylation and methionine and acetyl (protein N terminal) oxidation were used as the fixed modification and the variable modifications in the search criteria, respectively. The initial mass tolerances for precursor and fragment ions were set at 10 ppm and 0.02 Da, respectively [17]. UniProt human (uniprot_human_73940_20190731_iRT.fasta) and Biognosys' iRT peptides fasta (uploaded to the public repository) databases acted as references for DDA data retrieval.

### 2.6. Proteomic Analyses

After minimizing biases between experiments through median normalization, protein expression differences were then evaluated via a Student's *t*-test. Statistically significant differentially expressed proteins (DEPs) were defined using *p* < 0.05 and fold-change cut-offs of >1.5 and <0.667 (metabolite ratios >1.5 and <0.667 were classified as increased and decreased, respectively). Data normalization, DEPs, Gene Ontology (GO), and Kyoto Encyclopedia of Genes and Genomes (KEGG) analyses were performed in ‘Wu Kong' platform (URL: https://www.omicsolution.com/wkomics/main/) [18]. Protein-protein interaction (PPI) networks of DEPs were constructed using the Search Tool for the Retrieval of Interacting Genes (STRING), with a combined score above 0.4 indicating statistical significance. Betweenness centrality was measured using CytoNCA plugin in Cytoscape v3.9.1 for the hub gene screening [19, 20]. “Without weight” was set as the parameter. IPA (Ingenuity Systems, USA) was utilized to discuss disease and biological function, Tox function enrichment and upstream regulatory molecules/networks.

### 2.7. Statistical Processing

A normality test was performed on all data. Continuous variables of normal distribution are expressed as mean ± standard deviation, and categorical variables are given numbers (percentages). Independent Student's *t*-test, Fisher's exact test or the Chi-squared test explored the intergroup difference of characteristics. A *p* value <0.05 was the significance level. R Statistical Software (RStudio, Inc., Boston, MA, USA; version 1.0.153) performed statistical analyses and plotting.

## 3. Results

### 3.1. Identification of DEPs

**Figure 1:**
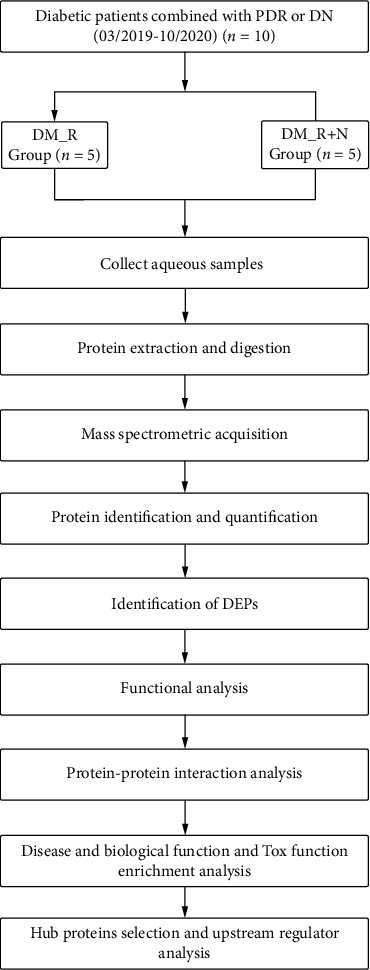
Research flowchart.

Figure 1 shows the workflow of our study.  Table 1 shows patients' clinical features: PDR (DM_R) and PDR and nephropathy (DM_R+N) patients, 5 cases each with corresponding average ages 45.6 ± 15.4 and 42.2 ± 8.1 years (*p* = 0.674; Table 1). The two groups were statistically similar regarding gender ratio, age, HbA1c, duration of diabetes, fasting blood glucose (FBG), and indication for surgery. Macroalbuminuria was present in the DM_R+N group (>300 mg/24 h).

**Figure 2:**
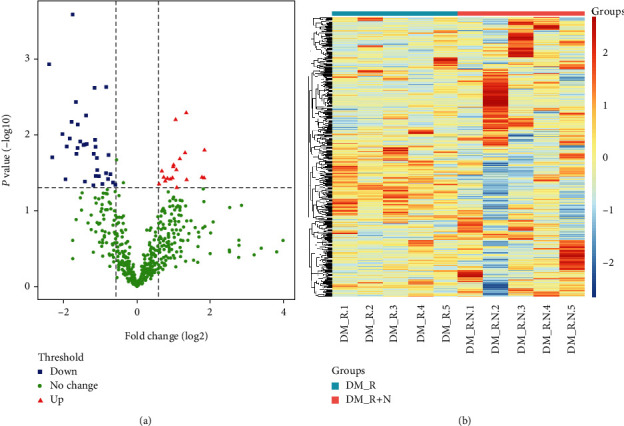
Differential expressed proteins analysis. (a) Valcano plot of the DEPs. (b) Heatmap of the DEPs.

Large-scale LC-MS/MS analysis of all gel bands retrieved 692 unique proteins (Table S1). Filter by 0.5 missing ratio in each group, and after filling by the global minimum method, 496 common to all cases were further studied, identifying 54 statistically significant DEPs, 19 that were upregulated and 35 that were downregulated statistically significant DEPs (volcano plot Figure 2(a), heatmap Figure 2(b)) (Table 2).

### 3.2. GO and KEGG Pathway Enrichment Analysis

**Figure 3:**
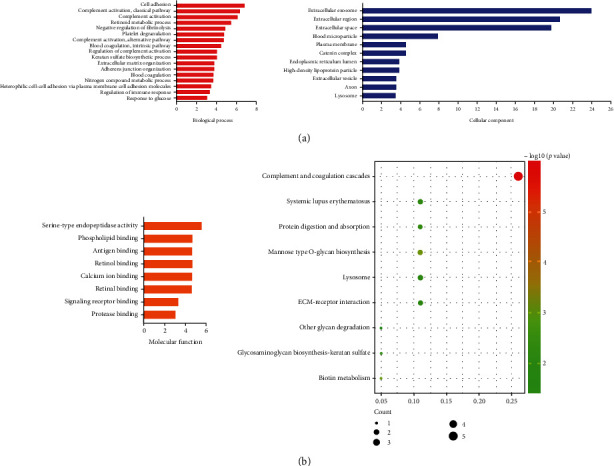
Functional enrichment pathway analysis. (a) Go enrichment. (b) KEGG enrichment.

DEPs GO was categorized as biological processes, cellular components, and molecular functions (Figure 3(a)). In the top 3 biological process group, DEPs were dominantly enriched in cell adhesion, complement activation, and classical pathway. DEPs in the top 3 cellular component group were primarily enriched in extracellular exosome/area/space. The top 3 molecular function group DEPs were dominantly enriched in cell adhesion, complement activation, and classical pathway. DEGs were predominantly enriched in complement and coagulation cascades, as indicated by KEGG enrichment analysis in Figure 3(b).

### 3.3. Protein Networks

**Figure 4:**
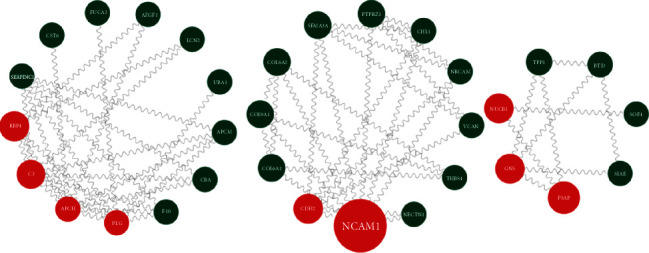
PPI network demonstrates the relationships between proteins.

To better understand the relationship between DEPs, we utilized the STRING database for PPI analysis. The PPI can be classified as either known interaction (curated databases and experimental determination of literature), predicted interaction (gene-neighborhood, gene fusion, and gene cooccurrence), or others (text mining, coexpression, and protein homology). See Table S2 for detailed information. There were 34 (63.0%) proteins interacting with other proteins in the 54 DEPs. Proteins with betweenness centrality above 10 include NCAM1, PLG, APOH, C3, PSAP, RBP4, CDH2, NUCB1, and GNS (Table 3, Figure 4).

Red indicated the differentially expressed proteins with betweenness centrality above 10; green indicated differentially expressed proteins with betweenness centrality below 10.

### 3.4. Disease and Biological Function and Tox Function Enrichment Analyses

We performed disease and biological function and Tox function enrichment analyses for 54 DEPs with the QIAGEN IPA (QIAGEN, USA; URL: http://www.qiagen.com/ingenuity).

As shown in Table 4, DEPs were enriched to 15 functional categories involving renal and urological disease with statistical significance, including renal vein thrombosis, migration, atypical hemolytic uremic syndrome (aHUS), failure, biotinidase deficiency, septic acute kidney injury, staghorn calculus, susceptibility to aHUS type 5, end stage renal disease (ESRD), aHUS, adhesion, acute tubular necrosis, C3 glomerulopathy, nephrosis, and membranoproliferative glomerulonephritis. The corresponding diseases or function annotations were renal vein thrombosis, migration of kidney cell lines, aHUS, failure of kidney, biotinidase deficiency, septic acute kidney injury, staghorn calculus, susceptibility to aHUS type 5, ESRD, acute phase aHUS, adhesion of kidney cell lines, acute tubular necrosis, C3 glomerulopathy, nephrosis, and membranoproliferative glomerulonephritis, respectively.

As shown in Table 5, DEPs were enriched to 11 Tox functional categories involving renal and urological system abnormalities with statistical significance. The specific diseases or functions annotation involved include failure of kidney, ESRD, acute tubular necrosis, C3 glomerulopathy, nephrosis, ischemic acute renal failure, membranoproliferative glomerulonephritis, steroid-dependent nephrotic syndrome, and IgA nephropathy.

DEPs involved in renal and urological system abnormalities include C3, F10, BTD, LCN2, PLG, SEMA3A, SERPINC1, CHL1, and RBP3. Among them, C3 and PLG were also the selected hub proteins in PPI networks of all DEPs with betweenness centrality above 10.

### 3.5. Analysis of Upstream Regulators

Upstream regulator analysis, in which IPA was used to statistically enrich upstream regulators, was used to identify candidate upstream regulators of proteins (Table S3).

Upstream factors statistically affecting C3 included IL1B, PPARA, TWIST1, Tnf receptor, estrogen receptor, EZH2, NR1H2, C3AR1, miR-291a-3p (and other miRNAs w/seed AAGUGCU), EHMT1, CD46, and IL6. Upstream factors statistically affecting PLG included JINK1/2, Jnk, Akt, and IL6.

## 4. Discussion

DN has become the prime culprit for ESRD in both the developed and developing countries [21, 22]. However, because of the complicated etiology of DN as well as ethnic differences, its molecular mechanism remains uncharacterized. Many DN patients are diagnosed late and are difficult to cure with conventional therapy [23], which largely underlies DN patients' adverse renal outcomes. Hence, there is an urgent need for potential markers that can facilitate early diagnosis and targeted therapies. Because DN often follows DR in the development of microvascular complications of diabetes, it is worth to set DR combined with DN as the observation group and DN as the control group to explore the AH proteins modulated by DN.

In the present study, proteins in AH samples of 5 patients with PDR combined DN and 5 patients with PDR were quantified, and the DEPs between two groups were identified. Then, GO and KEGG pathway enrichment were analyzed (Figures 3(a) and 3(b)). Moreover, PPI was analyzed and hub proteins were selected, including NCAM1, PLG, APOH, C3, PSAP, RBP4, CDH2, NUCB1, and GNS. To further search for hub proteins associated with renal and urological disease, IPA was used. C3 and PLG were selected as hub proteins associated with renal and urological disease.

C3 is central in complement system activation. The proteolytic cleavage of C3 by C3 convertases is the key reaction in both classical and alternative pathways of complement. Following activation, C3b can perform covalent binding to cell surface carbohydrates or immune aggregates through its reactive thioester. PLG can dissolve blood clot fibrin and functions as a proteolytic factor in embryonic development, tissue remodeling, neoplasm invasiveness, inflammation, and many other processes. It is also capable of activating urokinase-type plasminogen activators and collagenases, as well as complement zymogens like C1 and C5. And fibronectin and laminin cleavage results in cell detachment and apoptosis.

Several studies have found that C3 and PLG are essential in the pathogenesis of DN. Zhao et al. found C3 correlated negatively with annual estimated glomerular filtration rate decline [24]. Moreover, C3 has also been identified as possibly involved in diabetic tubulointerstitial injury by Zeng et al. [25]. And PLG was determined by Wang et al. to be critical in regulating the occurrence of DN [26]. Caseiro et al. analyzed alterations in urine proteomes in T1DM patients with and without complications (e.g., DR and DN) and identified that ephrin type-B receptor 4 and vitamin K-dependent protein Z were feasible markers for advanced T1DM complicated by DR or DN [27]. However, this study did not analyze the differentially coexpressed proteins between the DR combined DN group and DR group, nor did the betweenness centrality use to screen hub proteins, and IPA was not used to select proteins associated with renal and urological disease.

Advances in analytical techniques and database search programs have substantially supported the proteomic research on DR and DN. The identification of DEPs is the key to revealing specific pathological processes. Proteins of particular interest that occupy a place in the PDR combined DN/PDR DEP list may be the candidate biomarkers for early-stage DN [28]. The limitations of this work mainly lie in the following two aspects: one is the absence of verification of DEPs, for which orthogonal analyses, including western blotting, radioimmunoassay, Enzyme-Linked Immunosorbent Assays (ELISA), and immunohistochemistry should be carried out for validation [29, 30]; the other is the limited samples included that cannot contribute to a meaningful evaluation, requiring further research with expanded sample size.

## 5. Conclusion

This research, as far as we are aware, is the first to analyze the mechanism of progression of DN on the basis of PDR in AH samples. The findings offer novel insights into alterations of AH proteomes in PDR combined DN patients and further validation of key proteins previously found in other tissue samples such as urine and blood [25, 31]. The selected hub proteins may interfere with the regulation of PDR comorbid DN from PDR in AH, especially for C3 and PLG, but further work for validation of this hypothesis is warranted.

## Figures and Tables

**Table 1:** Subjects' characteristics^∗^.

Variables		DM_R group (*n* = 5)	DM_R+N group (*n* = 5)	*χ* ^2^/*t*	*p* ^†^
Age (y)		45.6 ± 15.4	42.2 ± 8.1	0.437	0.674
Male gender (%)		3 (60.0)	4 (80.0)	0.476	0.490
Right eye (number (%))		5 (100.0)	3 (60.0)	2.500	0.114
HbA1c (%)		8.1 ± 1.3	7.9 ± 0.4	0.329	0.751
Duration of diabetes (years)		9.2 ± 6.7	11.0 ± 5.0	0.615	0.556
Fasting blood glucose (mmol/L)		11.3 ± 6.0	7.4 ± 2.3	1.357	0.212
Indication for surgery					
	Vitreous hemorrhage	5 (100.0)	5 (100.0)		—
	Tractional retinal detachment	3 (60.0)	2 (40.0)	0.400	0.527

^∗^ Quantitative data and qualitative data were expressed as mean ± SD and number of people (%), respectively; ^†^*p* values refer to independent Student's *t*-test, Fisher's exact test, or the Chi-squared test exploring the difference of characteristics between groups.

**Table 2:** Significantly differentially expressed proteins.

	Description	p.t. test	Fold-change	Express
A0A0A0MT36	Immunoglobulin kappa variable 6D-21 (nonfunctional)(IGKV6D-21)	0.036955223	3.57463889	Upregulate
A0A0C4DH25	Immunoglobulin kappa variable 3D-20 (IGKV3D-20)	0.049493681	2.128066835	Upregulate
A0A286YEY1	Immunoglobulin heavy constant alpha 1 (IGHA1)	0.028949107	2.110253625	Upregulate
B4E1Z4	Complement factor B (ENSP00000410815)	0.026456377	1.992205397	Upregulate
E7ES19	Thrombospondin 4 (THBS4)	0.017307964	2.481397525	Upregulate
P00742	Coagulation factor X (F10)	0.037825362	1.773541552	Upregulate
P00747	Plasminogen (PLG)	0.036283396	1.661644313	Upregulate
P01008	Serpin family C member 1 (SERPINC1)	0.030104627	1.601734876	Upregulate
P01024	Complement C3 (C3)	0.04465175	1.521368772	Upregulate
P01602	Immunoglobulin kappa variable 1-5 (IGKV1-5)	0.025182741	2.001283426	Upregulate
P01709	Immunoglobulin lambda variable 2-8 (IGLV2-8)	0.040265165	1.711371053	Upregulate
P02749	Apolipoprotein H (APOH)	0.039317619	2.55723495	Upregulate
P02753	Retinol binding protein 4 (RBP4)	0.038694982	1.88456551	Upregulate
P07357	Complement C8 alpha chain (C8A)	0.037286856	1.950270583	Upregulate
P22314	Ubiquitin like modifier activating enzyme 1 (UBA1)	0.036426384	3.419798742	Upregulate
P25311	Alpha-2-glycoprotein 1, zinc-binding (AZGP1)	0.006282018	2.078922885	Upregulate
P80188	Lipocalin 2 (LCN2)	0.020689034	2.250249072	Upregulate
Q15828	Cystatin E/M (CST6)	0.015888421	3.585016053	Upregulate
Q5T123	SH3 domain binding glutamate rich protein like 3 (SH3BGRL3)	0.005080205	2.546679302	Upregulate
A0A087WX77	Neural cell adhesion molecule 1 (NCAM1)	0.013520081	0.362177114	Downregulate
A0A087X0M8	Cell adhesion molecule L1 like (CHL1)	0.002396942	0.447154673	Downregulate
A0A087X0S5	Collagen type VI alpha 1 chain (COL6A1)	0.002327337	0.556691701	Downregulate
A0A087X1J7	Glutathione peroxidase 3 (GPX3)	0.042604595	0.636518049	Downregulate
A0A2R8Y7U1	Tripeptidyl peptidase 1 (TPP1)	0.007284898	0.324649629	Downregulate
C9IZG4	cutA divalent cation tolerance homolog (CUTA)	0.014864747	0.323983214	Downregulate
C9JIZ6	Prosaposin (PSAP)	0.044621913	0.51799473	Downregulate
C9JYY6	Neuronal cell adhesion molecule (NRCAM)	0.011508226	0.450951245	Downregulate
E9PF17	Versican (VCAN)	0.038360004	0.257367031	Downregulate
F6S8M0	Glucosamine (N-acetyl)-6-sulfatase (GNS)	0.012224358	0.341371268	Downregulate
F6VDH7	ST13 Hsp70 interacting protein (ST13)	0.019827016	0.200673989	Downregulate
H0YF95	Seizure related 6 homolog (SEZ6)	0.013350489	0.371791006	Downregulate
K7ELL7	Protein kinase C substrate 80 K-H (PRKCSH)	0.017635437	0.43953481	Downregulate
O43505	Beta-1,4-glucuronyltransferase 1 (B4GAT1)	0.045900429	0.662871362	Downregulate
O60575	Serine peptidase inhibitor Kazal type 4 (SPINK4)	0.046274131	0.436778293	Downregulate
O75882	Attractin (ATRN)	0.032912143	0.603382567	Downregulate
O95445	Apolipoprotein M (APOM)	0.011195521	0.27929918	Downregulate
P10745	Retinol binding protein 3 (RBP3)	0.000256535	0.294947516	Downregulate
P12110	Collagen type VI alpha 2 chain (COL6A2)	0.041236514	0.371699968	Downregulate
P15291	Beta-1,4-galactosyltransferase 1 (B4GALT1)	0.03477554	0.458653103	Downregulate
P19022	Cadherin 2 (CDH2)	0.029216973	0.468621717	Downregulate
P20849	Collagen type IX alpha 1 chain (COL9A1)	0.00669932	0.287809307	Downregulate
P23471	Protein tyrosine phosphatase receptor type Z1 (PTPRZ1)	0.005534332	0.378623205	Downregulate
P43251	Biotinidase (BTD)	0.018418073	0.582873713	Downregulate
Q02818	Nucleobindin 1 (NUCB1)	0.035050498	0.482356964	Downregulate
Q14563	Semaphorin 3A (SEMA3A)	0.017746065	0.317266926	Downregulate
Q15223	NECTIN cell adhesion molecule 1 (NECTIN1)	0.001170444	0.188635993	Downregulate
Q15904	ATPase H+ transporting accessory protein 1 (ATP6AP1)	0.003694191	0.314344708	Downregulate
Q6UX71	Plexin domain containing 2 (PLXDC2)	0.031813223	0.559106065	Downregulate
Q8WXD2	Secretogranin III (SCG3)	0.014293057	0.455315443	Downregulate
Q96JP9	Cadherin related family member 1 (CDHR1)	0.014155971	0.262671062	Downregulate
Q99784	Olfactomedin 1 (OLFM1)	0.013202271	0.386150368	Downregulate
Q9BRK5	Stromal cell derived factor 4 (SDF4)	0.009752638	0.243429438	Downregulate
Q9BTY2	Alpha-L-fucosidase 2 (FUCA2)	0.038217313	0.576768404	Downregulate
Q9HAT2	Sialic acid acetylesterase (SIAE)	0.020162057	0.466031421	Downregulate

**Table 3:** Differentially expressed proteins with Betweenness centrality above 10.

	Proteins	Betweenness centrality
1	NCAM1	Betweenness: 62.5
2	PLG	Betweenness: 44.5
3	APOH	Betweenness: 40.833332
4	C3	Betweenness: 19.833334
5	PSAP	Betweenness: 16.0
6	RBP4	Betweenness: 11.0
7	CDH2	Betweenness: 10.166667
8	NUCB1	Betweenness: 10.0
9	GNS	Betweenness: 10.0

**Table 4:** Functional enrichment involving renal and urological disease for differentially expressed proteins.

Categories	Functions	Diseases or functions annotation	*p* value	Molecules	# molecules
Cardiovascular disorders, hematological diseases, organismal injuries and abnormalities, and renal and urological disease	Renal vein thrombosis	Renal vein thrombosis	0.0000364	F10, SERPINC1	2
Cellular movement and renal and urological system development and function	Migration	Migration of kidney cell lines	0.000108	C3, CHL1, F10, RBP3	4
Cardiovascular disorders, cell death and survival, connective tissue disorders, hematological diseases, organismal injuries and abnormalities, and renal and urological disease	Atypical hemolytic uremic syndrome	Atypical hemolytic uremic syndrome	0.000907	C3, PLG	2
Organismal injuries and abnormalities and renal and urological disease	Failure	Failure of kidney	0.00237	C3, F10, SERPINC1, LCN2	4
Developmental disorder, hereditary disorder, metabolic disease, organismal injuries and abnormalities, and renal and urological disease	Biotinidase deficiency	Biotinidase deficiency	0.00249	BTD	1
Organismal injuries and abnormalities and renal and urological disease	Septic acute kidney injury	Septic acute kidney injury	0.00249	LCN2	1
Organismal injuries and abnormalities and renal and urological disease	Staghorn calculus	Staghorn calculus	0.00249	PLG	1
Cardiovascular disorders, cell death and survival, connective tissue disorders, hematological diseases, organismal injuries and abnormalities, and renal and urological disease	Susceptibility to atypical hemolytic uremic syndrome type 5	Susceptibility to atypical hemolytic uremic syndrome type 5	0.00249	C3	1
Organismal injuries and abnormalities and renal and urological disease	End stage renal disease	End stage renal disease	0.00318	C3, F10, SERPINC1	3
Cardiovascular diseases, cell death and survival, connective tissue disorders, hematological diseases, organismal injuries and abnormalities, and renal and urological disease	Atypical hemolytic uremic syndrome	Acute phase atypical hemolytic uremic syndrome	0.00497	C3	1
Cell-to-cell signaling and interaction and renal and urological system development and function	Adhesion	Adhesion of kidney cell lines	0.00664	C3, F10	2
Organismal injuries and abnormalities and renal and urological disease	Acute tubular necrosis	Acute tubular necrosis	0.00745	LCN2	1
Organismal injuries and abnormalities and renal and urological disease	C3 glomerulopathy	C3 glomerulopathy	0.00745	C3	1
Organismal injuries and abnormalities and renal and urological disease	Nephrosis	Nephrosis	0.00861	C3, F10, SERPINC1	3
Immunological disease, inflammatory disease, inflammatory response, organismal injuries and abnormalities, andrenal and urological disease	Membranoproliferative glomerulonephritis	Membranoproliferative glomerulonephritis	0.00992	C3	1

**Table 5:** Tox functional categories involving renal and urological system abnormalities for differentially expressed proteins.

Categories	Functions	Diseases or functions annotation	*p* value	Molecules	# molecules
Kidney failure	Failure	Failure of kidney	0.00237	C3,F10,SERPINC1,LCN2	4
Kidney failure	End stage renal disease	End stage renal disease	0.00318	C3,F10,SERPINC1	3
Kidney failure	Acute tubular necrosis	Acute tubular necrosis	0.00745	LCN2	1
Glomerular injury	C3 glomerulopathy	C3 glomerulopathy	0.00745	C3	1
Nephrosis	Nephrosis	Nephrosis	0.00861	C3,F10,SERPINC1	3
Kidney failure and renal damage	Ischemic acute renal failure	Ischemic acute renal failure	0.00992	LCN2	1
Glomerular injury, renal inflammation, renal nephritis	Membranoproliferative glomerulonephritis	Membranoproliferative glomerulonephritis	0.00992	C3	1
Nephrosis	Steroid-dependent nephrotic syndrome	Steroid-dependent nephrotic syndrome	0.0295	C3	1
Glomerular injury, renal inflammation, and renal nephritis	IgA nephropathy	IgA nephropathy	0.0882	C3	1
Glomerular injury, renal inflammation, and renal nephritis	Lupus nephritis	Lupus nephritis	0.412	C3	1
Renal necrosis/cell death	Apoptosis	Apoptosis of kidney cell lines	0.478	RBP4	1

## Data Availability

The datasets generated during the current study are available from the corresponding author on reasonable request.

## Supplementary Materials

Supplementary 1 Table S1: Unique proteins were detected by the large-scale LC-MS/MS analysis.

Supplementary 2 Table S2: Protein-protein interaction analysis results using the STRING database.

Supplementary 3 Table S3: Statistically significant upstream regulators from IPA.
