# Supplementary material for: Pursuing Diabetic Nephropathy through Aqueous Humor Proteomics Analysis
Source: Oxid Med Cell Longev. 2022 Sep 29;2022:5945828. doi: 10.1155/2022/5945828 (PMC9537621; doi:10.1155/2022/5945828)
Supplement: Supplementary 1 — Table S1: Unique proteins were detected by the large-scale LC-MS/MS analysis. [file 5945828.f1.doc]

**Table S1. Unique proteins were detected by the large-scale LC-MS/MS analysis**

| **PG** | DM_R 1 | DM_R 2 | DM_R 3 | DM_R 4 | DM_R 5 | DM_R+N 1 | DM_R+N 2 | DM_R+N 3 | DM_R+N 4 | DM_R+N 5 |
| --- | --- | --- | --- | --- | --- | --- | --- | --- | --- | --- |
| A0A024R0T9 | 24823.50781 | 65098.42188 | 9170.553711 | 6560.387207 | 33762.59766 | 22390.17578 | 0 | 54969.625 | 30780.19531 | 0 |
| A0A075B6I0 | 9147.970703 | 43147.03906 | 45855.55469 | 0 | 11693.23535 | 60401.37891 | 475999.5625 | 0 | 9301.074219 | 0 |
| A0A075B6I9 | 4132.687012 | 7579.480957 | 23257.11328 | 4851.354492 | 6822.955078 | 0 | 43969.43359 | 23176.46484 | 0 | 17778.71484 |
| A0A075B6J9 | 7686.951172 | 18516.86523 | 12799.87891 | 9518.90332 | 11267.0127 | 19212.60352 | 61773.92969 | 46156.06641 | 9997.804688 | 27810.98047 |
| A0A075B6K4 | 61448.71484 | 0 | 9633.612305 | 15172.66504 | 0 | 39120.55469 | 191043.4063 | 9978.125 | 45377.67969 | 0 |
| A0A075B6K5 | 66868.03125 | 119432.3672 | 110753.9375 | 35584.59766 | 91593.82813 | 47108.76563 | 227961.2656 | 380840.5313 | 50318.85156 | 101615.9688 |
| A0A075B6P5 | 73031.71875 | 101119.1484 | 103751.7422 | 150722.1406 | 70281.53125 | 209740.1563 | 49098.12109 | 393229.5313 | 99166.50781 | 270655.8438 |
| A0A075B6R2 | 38476.75391 | 4397.453125 | 0 | 6153.054199 | 0 | 22707.375 | 29405.01563 | 26205.89453 | 5294.023438 | 5241.343262 |
| A0A075B6R9 | 2925.509521 | 14042.66211 | 18593.83398 | 23017.86523 | 5226.237305 | 34301.72656 | 0 | 43977.54688 | 9255.432617 | 9842.707031 |
| A0A075B6S2 | 250833.7813 | 412968.6875 | 650731.0625 | 277354.125 | 494329.9375 | 284128.6875 | 1702405.5 | 753041.5 | 339049.6875 | 693512.9375 |
| A0A075B6S5 | 1367.829712 | 4640.032227 | 10135.55273 | 4828.585938 | 0 | 5636.394531 | 0 | 15455.68848 | 34674.11328 | 14595.34863 |
| A0A075B7B8 | 43166.08984 | 28740.36133 | 16056.60645 | 28393.38867 | 38110.23828 | 19149.91602 | 150925.375 | 37963.08984 | 17884.70898 | 69229.40625 |
| A0A075B7D0 | 503987.125 | 139571.6094 | 347909.1875 | 100408.0078 | 328066.9375 | 254968.9219 | 791455.1875 | 605148.875 | 222171.6094 | 425144.8125 |
| A0A075B7D8 | 2446.627686 | 2846.258057 | 5899.844727 | 6381.640137 | 0 | 0 | 0 | 5486.351074 | 0 | 0 |
| A0A087WSY4 | 10940.09961 | 40582.14063 | 42486.22656 | 12562.78223 | 14485.50781 | 24742.47266 | 103233.9375 | 69081.88281 | 31779.74219 | 25696.58203 |
| A0A087WSY5 | 28254.62109 | 26970.80469 | 22098.8125 | 24699.72266 | 27768.38477 | 22567.71094 | 81397.21875 | 30786.78516 | 32459.96875 | 36698.17188 |
| A0A087WSY6 | 100473.0781 | 65249.32031 | 155633.6563 | 121891.6406 | 81848.13281 | 219109.7344 | 141389.875 | 231710.9844 | 124254.1797 | 235126.7813 |
| A0A087WTA8 | 10254.91992 | 6119.164551 | 6341.52832 | 5634.540039 | 8410.125977 | 13084.85547 | 16950.75 | 3465.681152 | 5714.089355 | 3731.305176 |
| A0A087WTY6 | 111530 | 93332.49219 | 111654.1953 | 49290.58203 | 71605.875 | 35917.18359 | 44976.80469 | 26875.4375 | 42928.58594 | 112598.9297 |
| A0A087WUD8 | 12059.9209 | 10848.31836 | 4494.940918 | 4463.32666 | 3074.716309 | 3245.6875 | 0 | 7907.845215 | 6995.246094 | 0 |
| A0A087WVQ9 | 2993.706055 | 3568.600098 | 3684.281494 | 5260.165527 | 6991.04541 | 18429.65039 | 30855.58789 | 0 | 0 | 0 |
| A0A087WVR1 | 7148.316406 | 6515.274414 | 3192.75 | 4205.442871 | 0 | 14770.12305 | 0 | 6711.308105 | 7210.562012 | 0 |
| A0A087WX77 | 10454.9375 | 8538.65332 | 11914.56445 | 8640.856445 | 10318.03223 | 4743.972168 | 0 | 7529.10791 | 8171.167969 | 0 |
| A0A087WXI5 | 1757.610352 | 4739.702148 | 1095.266113 | 921.0395508 | 1153.971802 | 0 | 0 | 0 | 815.3262329 | 2598.14502 |
| A0A087WXX2 | 421.8819885 | 1392.752441 | 845.2376709 | 0 | 0 | 0 | 0 | 0 | 0 | 1207.374512 |
| A0A087WYG6 | 8448.856445 | 4031.241211 | 8109.411621 | 0 | 0 | 1411.712769 | 0 | 0 | 0 | 0 |
| A0A087WYL5 | 1141.581787 | 2235.055664 | 3205.958252 | 0 | 0 | 0 | 0 | 0 | 0 | 0 |
| A0A087WZM2 | 6660.195801 | 3456.205322 | 3631.432617 | 3472.177979 | 3519.993164 | 3114.648682 | 0 | 0 | 3039.881104 | 5391.529785 |
| A0A087X054 | 3331.493164 | 2024.269409 | 5241.041016 | 0 | 5109.521973 | 0 | 0 | 0 | 0 | 0 |
| A0A087X0D5 | 5531.353516 | 3134.102295 | 4184.638184 | 438.6916809 | 674.9263916 | 1589.353394 | 0 | 0 | 3092.360596 | 4997.255371 |
| A0A087X0I0 | 2091.952637 | 680.8703613 | 9093.795898 | 18699.35547 | 32834.84375 | 292.3002014 | 0 | 0 | 0 | 7685.983398 |
| A0A087X0K0 | 4121.160645 | 1558.637207 | 1168.860962 | 1673.072388 | 2571.533936 | 5786.998047 | 16722.34766 | 2959.348145 | 8046.38916 | 0 |
| A0A087X0M8 | 23376.91406 | 17429.66211 | 26374.19922 | 26262.78125 | 14051.68945 | 8827.605469 | 9401.617188 | 18596.41602 | 12927.60547 | 8561.015625 |
| A0A087X0S5 | 29836.85938 | 21851.29688 | 26267.12109 | 31660.17188 | 18873.96094 | 14419.49316 | 27655.80273 | 12647.92188 | 16923.7832 | 18432.42188 |
| A0A087X0T8 | 11748.30664 | 11545.82031 | 13031.98633 | 2810.125977 | 6646.018555 | 0 | 0 | 0 | 5937.719727 | 0 |
| A0A087X191 | 952.815979 | 624.519043 | 606.8564453 | 0 | 0 | 0 | 0 | 0 | 0 | 0 |
| A0A087X1J7 | 409815.4688 | 422060.5938 | 582159.9375 | 535393.125 | 306737.7813 | 227389.8906 | 439612 | 254946.0938 | 351889.9688 | 470562.4375 |
| A0A087X1L8 | 9386.916992 | 12173.01074 | 9845.924805 | 17203.36328 | 7866.986328 | 13069.31934 | 0 | 13676.95117 | 8884.333984 | 19113.86914 |
| A0A087X208 | 10807.18652 | 6413.600098 | 10206.63379 | 6114.246094 | 6241.590332 | 10262.84668 | 21074.88672 | 8423.964844 | 2560.694336 | 8397.654297 |
| A0A087X232 | 102870.3672 | 97840.9375 | 83390.78125 | 93663.41406 | 114908.875 | 170056.7344 | 161444.5938 | 105810.9219 | 77037 | 220800.8125 |
| A0A087X2B5 | 2731.241943 | 2629.466797 | 3321.339111 | 0 | 0 | 0 | 0 | 0 | 0 | 0 |
| A0A096LPE2 | 10376.61523 | 32794.78906 | 12195.41211 | 7280.431641 | 27310.1543 | 51653.80469 | 101938.1484 | 91679.40625 | 32350.62109 | 12786.61816 |
| A0A0A0MR55 | 1384.428223 | 1172.168213 | 3027.364746 | 0 | 0 | 0 | 0 | 0 | 0 | 0 |
| A0A0A0MRJ7 | 26494.32031 | 12987.56348 | 18584.85742 | 11622.20313 | 7444.259766 | 28521.55469 | 18784.91797 | 13270.93164 | 13241.53906 | 7380.276367 |
| A0A0A0MRZ8 | 895514.8125 | 955710.4375 | 1232715.625 | 710538.5625 | 1038990.125 | 1460191.625 | 4837082 | 3078210.75 | 1096699.125 | 1658530.125 |
| A0A0A0MS08 | 16022949 | 11625264 | 20177792 | 9388941 | 21998552 | 19610066 | 74081184 | 52176060 | 16970642 | 25120474 |
| A0A0A0MS09 | 9497.292969 | 0 | 0 | 11791.42285 | 5784.919434 | 8036.652344 | 48201.51563 | 0 | 0 | 0 |
| A0A0A0MS15 | 12433.09082 | 24395.05469 | 73614.04688 | 39925.86328 | 45263.66406 | 20563.30859 | 11698.71289 | 56975.16797 | 30009.30859 | 106552.8438 |
| A0A0A0MSA7 | 38898.375 | 9524.785156 | 22990.32227 | 1588894 | 19996.38867 | 370777.625 | 1346618.75 | 23524.11914 | 36032.26953 | 83367.66406 |
| A0A0A0MSA9 | 5866.291504 | 5469.213867 | 4163.243652 | 6446.578613 | 3419.397949 | 7175.490234 | 0 | 10424.63965 | 4404.927734 | 7746.272461 |
| A0A0A0MSQ0 | 6839.570313 | 3117.687744 | 5201.206055 | 9832.010742 | 12748.02832 | 9552.723633 | 0 | 25898.44727 | 9568.736328 | 14142.59766 |
| A0A0A0MSV6 | 18379.28711 | 20536.26172 | 14267.71582 | 8349.673828 | 11184.06738 | 10469.08496 | 38259.19922 | 16032.74805 | 11079.25391 | 8403.887695 |
| A0A0A0MT36 | 13422.52832 | 5102.88623 | 13489.2168 | 6792.684082 | 8821.080078 | 31909.38672 | 48865.67578 | 88084.60938 | 21411.49414 | 30732.40234 |
| A0A0A0MT69 | 5746.547363 | 0 | 0 | 0 | 0 | 13841.51563 | 17106.66602 | 0 | 1935.962891 | 0 |
| A0A0A0MT89 | 7329.894531 | 6460.252441 | 26152.98047 | 10043.66309 | 11485.06934 | 18347.1582 | 37988.07031 | 35091.87109 | 18774.86914 | 18094.94727 |
| A0A0A0MTI5 | 0 | 1595.441528 | 460.7740173 | 479.0915833 | 0 | 0 | 0 | 0 | 0 | 0 |
| A0A0A0MTS2 | 834.3463135 | 1120.777222 | 422.4475403 | 1501.964966 | 1621.334961 | 0 | 0 | 0 | 0 | 0 |
| A0A0B4J1U7 | 78709.14844 | 131805.7188 | 140552 | 162200.9375 | 102258.4375 | 143753.9688 | 398799.8125 | 228855.9844 | 144132.875 | 141380.875 |
| A0A0B4J1V0 | 7201.686523 | 8052.267578 | 11823.62891 | 3642.749756 | 7560.452148 | 0 | 9068.621094 | 3195.335693 | 4134.137207 | 12902.85938 |
| A0A0B4J1V2 | 5551.114258 | 13096.86523 | 18577.68359 | 15254.54492 | 7179.078613 | 13279.6709 | 0 | 31134.38867 | 26840.17383 | 43535.49219 |
| A0A0B4J1V6 | 3979.222412 | 10516.08301 | 5237.978516 | 2152.047852 | 3931.527588 | 3125.791504 | 22160.0293 | 0 | 0 | 8187.804688 |
| A0A0B4J1X5 | 585769.4375 | 485768.9688 | 664551.8125 | 640465.0625 | 519381.375 | 765164.6875 | 1445198 | 999386.75 | 366978.75 | 2446481 |
| A0A0B4J1Y8 | 7408.836426 | 33126.22656 | 30289.86914 | 5196.811523 | 12539.08105 | 19670.94336 | 44041.87109 | 24978.11914 | 8611.010742 | 7298.453613 |
| A0A0B4J1Y9 | 149940.8906 | 234761.0469 | 345840.7188 | 217210.4063 | 235058.2031 | 281503.1875 | 1171220 | 753973.4375 | 222738.3594 | 325114.25 |
| A0A0B4J231 | 1214981 | 990542.375 | 1024618.563 | 827309.375 | 1238386.25 | 1079005.25 | 2536144 | 3822456.5 | 1549216 | 1730507.5 |
| A0A0B4J2B5 | 633360.8125 | 1254410.625 | 969044.25 | 520578.8438 | 1407833.25 | 11505491 | 3010781 | 2738084.75 | 1098166.375 | 3344542 |
| A0A0C4DFP6 | 53601.34375 | 85697.53906 | 54433.00391 | 111186.2031 | 54398.45703 | 128024.5469 | 87000.17969 | 79540.11719 | 42954.60156 | 217223.5625 |
| A0A0C4DGL1 | 916.6647949 | 1419.175171 | 2343.378906 | 3069.226318 | 0 | 0 | 0 | 0 | 744.7032471 | 0 |
| A0A0C4DGN2 | 0 | 1404.683716 | 2700.216064 | 0 | 10501.0498 | 11393.78027 | 17015.78125 | 8795.275391 | 8177.382813 | 0 |
| A0A0C4DGV8 | 0 | 3485.936768 | 8550.454102 | 8617.008789 | 6601.946289 | 4786.825684 | 38271.47656 | 10003.1123 | 15217.73438 | 10009.06836 |
| A0A0C4DH24 | 0 | 7396.823242 | 10493.31641 | 8542.685547 | 0 | 0 | 44270.60547 | 10195.11035 | 0 | 9224.546875 |
| A0A0C4DH25 | 279852.4688 | 279173.6563 | 465149 | 278776.1875 | 347511.5 | 561268.5625 | 1498740.625 | 1600395.75 | 526507.4375 | 553586.0625 |
| A0A0C4DH29 | 1509.157471 | 2269.72583 | 5614.338867 | 4430.060059 | 5657.139648 | 0 | 0 | 0 | 3602.582275 | 4869.097656 |
| A0A0C4DH34 | 23958.30469 | 11154.51172 | 9024.924805 | 8068.336426 | 21331.20117 | 13478.67871 | 150363.125 | 35752.36328 | 37263.92188 | 23156.00781 |
| A0A0C4DH35 | 16396.88086 | 16373.33594 | 15141.3623 | 13244.59961 | 19406.17578 | 11493.99805 | 57903.81641 | 14525.3418 | 0 | 40175.78125 |
| A0A0C4DH36 | 6796.577148 | 3413.024902 | 8778.461914 | 4895.565918 | 12861.94141 | 6581.601563 | 41100.48438 | 9676.664063 | 2063.385986 | 7732.138184 |
| A0A0C4DH38 | 18480.4668 | 23622.18359 | 33399.29297 | 12573.91211 | 19027.89844 | 11382.83203 | 37994.64844 | 35354.77734 | 11277.21289 | 25121.19727 |
| A0A0C4DH41 | 47848.14453 | 35096.15625 | 81696.65625 | 19756.97461 | 27438.72461 | 21484.08984 | 20994.83789 | 44900.41406 | 30091.29297 | 66382.03125 |
| A0A0C4DH43 | 334077.4375 | 180728.5 | 451524.875 | 14536.82031 | 193189.8594 | 169968.6094 | 0 | 85045.92188 | 238160.8281 | 446791.375 |
| A0A0C4DH72 | 3575.75708 | 3927.020508 | 6687.351563 | 3194.731201 | 7989.330078 | 6947.966309 | 0 | 11553.15918 | 0 | 9422.467773 |
| A0A0G2JIW1 | 2526.095947 | 4207.817871 | 1895.113525 | 6329.585938 | 0 | 0 | 0 | 0 | 0 | 0 |
| A0A0G2JRQ6 | 48682.42578 | 83832.48438 | 99981.24219 | 35588.875 | 28194.43945 | 117404.3984 | 150256.5625 | 159506.5 | 29836.64648 | 53273.64453 |
| A0A0G2JS06 | 4358.04248 | 2801.413818 | 20742.38672 | 0 | 33240.92578 | 6207.623535 | 0 | 23359.60742 | 0 | 17135.52539 |
| A0A0J9YY99 | 98325.98438 | 84155.08594 | 79817.42969 | 35875.44141 | 41511.48047 | 109525.9688 | 380606.7188 | 90874.52344 | 34506.20313 | 220536.4844 |
| A0A0S2Z4L3 | 68267.49219 | 49390.08203 | 64566.54297 | 62534.67578 | 54837.70703 | 68198.53125 | 49094.27344 | 94584.98438 | 42420.03906 | 37273.97656 |
| A0A0U1RQC5 | 3599.643555 | 4084.88623 | 5754.234863 | 2359.845459 | 6506.020996 | 10887.82617 | 0 | 0 | 4265.836426 | 19759.99219 |
| A0A0U1RR20 | 2318.632324 | 1825.33667 | 3993.425049 | 3898.393066 | 0 | 5418.730469 | 31525.50781 | 7075.98584 | 1442.32251 | 3010.031494 |
| A0A140T912 | 23536.37891 | 8595.632813 | 10920.24805 | 3363.571289 | 10744.72168 | 1587.847778 | 0 | 0 | 12417.8623 | 5499.840332 |
| A0A1B0GTM3 | 4639.556641 | 6605.569336 | 6863.524414 | 4216.81543 | 6699.376465 | 12943.64453 | 14102.9082 | 0 | 0 | 0 |
| A0A1B0GUU9 | 96243.19531 | 173588.8281 | 50789.59766 | 23198.39258 | 259594.625 | 50664.28516 | 31411.47656 | 65723.26563 | 63901.27734 | 287617.7188 |
| A0A1B0GV23 | 685134.75 | 334073.1563 | 521281.4063 | 264512.7188 | 314277.8438 | 575798.125 | 330385.3125 | 277444.0625 | 280889.0625 | 137977.1563 |
| A0A1B0GV53 | 18261.57031 | 6647.943359 | 16404.19336 | 5206.949219 | 7916.962891 | 19587.21094 | 0 | 8801.585938 | 6403.679199 | 0 |
| A0A1B0GVB9 | 4119.061523 | 2963.847168 | 5162.150879 | 32569.95898 | 3517.877197 | 96737.89063 | 16623.37891 | 2835.365479 | 95039.58594 | 98729.84375 |
| A0A1W2PNW4 | 0 | 2917.696777 | 7794.393555 | 0 | 320.4354553 | 0 | 0 | 483684.4375 | 570.0077515 | 0 |
| A0A1W2PQB1 | 3596.22168 | 5139.240234 | 5159.170898 | 5850.00293 | 7466.631836 | 4726.910645 | 0 | 8921.251953 | 6358.525391 | 11452.25586 |
| A0A286YES1 | 58594.78516 | 71491.13281 | 279001.6563 | 33668.15625 | 131456.375 | 70480.15625 | 751697.3125 | 101282.1641 | 128698.0469 | 107329.2188 |
| A0A286YEY1 | 1193805 | 1309973.25 | 2378415.5 | 1641560 | 1812406.375 | 2272406.75 | 7904897.5 | 6389614.5 | 4137483 | 2667652 |
| A0A286YEY4 | 3217880.5 | 5488802 | 8170869.5 | 7282240.5 | 6248641 | 8616536 | 38703552 | 20214918 | 5775215.5 | 10853020 |
| A0A286YEY5 | 928499.4375 | 1116110.125 | 1992665.5 | 1314500.25 | 968122.6875 | 1608571.875 | 3112613.75 | 4618146 | 3311510.5 | 1998756.625 |
| A0A286YFF7 | 8195.189453 | 3750.566895 | 5787.795898 | 0 | 0 | 6814.358398 | 0 | 0 | 4273.154785 | 0 |
| A0A286YFJ8 | 103319.9531 | 60369.96094 | 107749.6406 | 46035.91406 | 136583.75 | 33966.28516 | 18781.76758 | 256902.6094 | 220519.4688 | 133652.3594 |
| A0A2Q2TTZ9 | 141115.6094 | 214500.1094 | 242639.25 | 101493.8984 | 187684.8125 | 95506.10156 | 425862.2813 | 272523.875 | 151456.9375 | 238113.1875 |
| A0A2R8Y3M9 | 256652.2656 | 216638.1719 | 282202.125 | 308803.1563 | 177185.0156 | 237057.6563 | 393935.0313 | 297282.625 | 257764.7344 | 271773.5313 |
| A0A2R8Y3T0 | 7411.690918 | 16949.79102 | 11550.41406 | 0 | 13513.13965 | 21478.68945 | 56017.0625 | 19437.02148 | 13746.93164 | 9693.202148 |
| A0A2R8Y430 | 0 | 1140.187256 | 2046.412231 | 2764.82666 | 1736.394165 | 0 | 0 | 0 | 2287.643555 | 2424.789551 |
| A0A2R8Y7U1 | 18135.14648 | 17295.74609 | 14175 | 9198.016602 | 8297.338867 | 11471.04688 | 0 | 0 | 4477.387207 | 7908.481445 |
| A0A2R8Y7X9 | 4540.35498 | 991.8373413 | 317.0927429 | 3117.859619 | 801.7179565 | 0 | 0 | 0 | 3992.828613 | 0 |
| A0A2R8YEC9 | 3558.005371 | 2955.969727 | 4129.432617 | 0 | 3099.190674 | 5834.745605 | 0 | 0 | 3504.62207 | 5341.12207 |
| A0A2U3TZL5 | 21405.24805 | 5391.17627 | 4250.160156 | 24951.74219 | 14064.12695 | 51054.07813 | 0 | 78996.42188 | 23644.93164 | 14129.35254 |
| A0A2U3U034 | 0 | 2599.544922 | 3875.522949 | 1663.248535 | 0 | 0 | 0 | 0 | 0 | 0 |
| A0A3B3IQ51 | 4061.145996 | 13684.16992 | 8181.478516 | 7542.699219 | 3644.314941 | 14797.26172 | 25220.79102 | 13208.33301 | 5809.577148 | 5546.669434 |
| A0A3B3IRN5 | 49443.60938 | 3946.018555 | 2786.290039 | 234097.8594 | 7397.848145 | 994609.5 | 6480765 | 6226.218262 | 431462.5938 | 3770.812988 |
| A0A3B3ISD1 | 0 | 966.5133057 | 2421.517822 | 801.8344727 | 0 | 0 | 0 | 0 | 0 | 0 |
| A0A3B3ISR2 | 49495.19531 | 26804.73047 | 20138 | 29118.79688 | 35412.35938 | 52931.82813 | 18725.23242 | 23571.13086 | 21463.2793 | 44723.04297 |
| A0A3B3ISS6 | 29624.68359 | 34659.80078 | 31509.86719 | 53180.34375 | 16190.34473 | 45233.75781 | 0 | 38380.89063 | 16875.89063 | 68022.22656 |
| A0A3B3IT96 | 7035.771973 | 7944.968262 | 4224.354492 | 5873.068359 | 1882.835449 | 0 | 0 | 0 | 3403.076904 | 6565.055664 |
| A0A3B3ITY6 | 67341.21094 | 39468.39844 | 42619.45313 | 54432.45313 | 21333.04688 | 25991.44922 | 0 | 17062.59961 | 21173.8418 | 54259.42188 |
| A0A3B3IUC0 | 1591.381104 | 0 | 447.1805115 | 968.3120117 | 0 | 0 | 0 | 0 | 0 | 0 |
| A0A3B3IUD5 | 15812.26563 | 15539.34863 | 21535.45703 | 17274.43164 | 15701.36426 | 17075.82227 | 42711.69531 | 8496.801758 | 16233.24121 | 24063.5625 |
| A0A3B3IUE0 | 45379.59375 | 60250.78906 | 51886.52344 | 78731.45313 | 32707.88086 | 58175.78516 | 44517.60938 | 36955.89063 | 30620.19531 | 74941 |
| A0AVL1 | 3813.987305 | 1229.85022 | 749.6547852 | 0 | 352.898468 | 0 | 0 | 0 | 0 | 0 |
| A2A2V1 | 4603.16748 | 6041.357422 | 7620.214355 | 4992.399414 | 0 | 0 | 0 | 0 | 0 | 4618.677246 |
| A2A3C1 | 5343.397461 | 4431.660645 | 7045.435059 | 7152.76709 | 0 | 0 | 0 | 0 | 2351.545898 | 0 |
| A6NGN9 | 0 | 2582.439209 | 1430.55188 | 16482.77734 | 38779.01953 | 0 | 0 | 0 | 0 | 0 |
| A8MVZ9 | 7509.336914 | 5713.563477 | 4590.978027 | 6394.098145 | 5812.773438 | 23406.8125 | 0 | 4405.545898 | 6835.879883 | 5375.515137 |
| A8MXB9 | 0 | 1003.625854 | 544.7512817 | 3026.429199 | 470.5123596 | 0 | 0 | 0 | 1 | 0 |
| B0QYH4 | 3537.620117 | 4900.326172 | 6289.095703 | 3137.64917 | 3389.921387 | 0 | 0 | 0 | 0 | 0 |
| B0YIW2 | 71582.90625 | 150286.125 | 35714.5625 | 26128.35742 | 57809.07031 | 46463.91797 | 36975.58594 | 161324.7813 | 61156.42188 | 0 |
| B0YJC4 | 2194.188477 | 832.2783813 | 2001.002441 | 721.5753784 | 972.8051758 | 0 | 0 | 1561.4823 | 1523.46875 | 0 |
| B1AHL2 | 17746.91602 | 0 | 0 | 10493.64746 | 0 | 15429.91406 | 0 | 9904.105469 | 9099.167969 | 0 |
| B1AP13 | 1397.383545 | 1201.403564 | 1314.248047 | 1621.121704 | 0 | 0 | 0 | 0 | 0 | 0 |
| B4DV12 | 5221.054688 | 5842.918945 | 6620.626953 | 3839.103027 | 6845.470703 | 0 | 0 | 0 | 6790.11377 | 11668.66211 |
| B4E1Z4 | 351706.2813 | 405999.9688 | 368217.8125 | 329350.8125 | 483814.5 | 663641.5 | 2208729.25 | 1041419.438 | 789098.3125 | 622728.625 |
| B7WNR0 | 64813.28906 | 53040.61328 | 74382.65625 | 79379.57813 | 82514.39063 | 16229.56543 | 24974.85156 | 71130.125 | 58189.90234 | 193759.3125 |
| B7Z4G8 | 23383.13477 | 27015.05469 | 30266.80078 | 27393.41211 | 22060.32227 | 14736.52832 | 51180.375 | 24900.29883 | 13051.37207 | 0 |
| B8ZZ51 | 7552.467773 | 7898.39209 | 11163.40039 | 10661.98926 | 6132.922852 | 17408.59375 | 0 | 0 | 6272.813477 | 6771.308594 |
| C9IZ46 | 28408.54688 | 28994.00977 | 7708.023926 | 34660.31641 | 31777.53516 | 19570.73438 | 31054.13672 | 37248.97266 | 0 | 46620 |
| C9IZG4 | 18242.79297 | 38086.04688 | 38650.49609 | 29163.72266 | 12617.22168 | 14550.48242 | 0 | 13823.80859 | 12315.83008 | 9402.566406 |
| C9J2H1 | 45583.07422 | 45871.33594 | 43979.51172 | 35138.36719 | 22737.17773 | 39504.60938 | 73162.46094 | 32928.14063 | 30517.3418 | 41151.96484 |
| C9J4D3 | 718.3764648 | 1474.835205 | 1340.689209 | 462.8686523 | 0 | 0 | 0 | 1440.242065 | 0 | 0 |
| C9J6G4 | 10896.34473 | 3286.621094 | 10514.87207 | 14985.10645 | 7283.029297 | 13890.93262 | 0 | 0 | 5521.568359 | 17442.85547 |
| C9J8S2 | 27548.2207 | 21602.9043 | 37782.55078 | 13997.03906 | 15834.55273 | 47648.87891 | 53816.89844 | 47123.82813 | 20590.29297 | 27922.77539 |
| C9JB90 | 29681.72852 | 104029.8906 | 0 | 115073.6094 | 13995.1875 | 0 | 0 | 0 | 0 | 0 |
| C9JC84 | 40426.50391 | 78960.92969 | 36965.41406 | 25283.64844 | 52332.77734 | 29990.91016 | 61627.39063 | 341133.5625 | 121502.9141 | 6845.429199 |
| C9JF17 | 369712.5 | 259664.5938 | 152052.8438 | 381122.9063 | 408800.1875 | 690363.1875 | 1128875.875 | 384908.0625 | 209182.1563 | 470967.8125 |
| C9JGG1 | 1377.499878 | 1015.000488 | 1510.015137 | 0 | 0 | 2325.008789 | 0 | 0 | 0 | 0 |
| C9JHR8 | 4560.275879 | 3290.221191 | 21524.69727 | 32288.36523 | 51483.79297 | 1907.396362 | 173231.8281 | 48535.85938 | 2699.492188 | 126404.9219 |
| C9JIZ6 | 101378.8359 | 93768.48438 | 84351.0625 | 75122.96875 | 40419.375 | 47952.78125 | 24707.69727 | 43807.97656 | 27507.59766 | 92834.75 |
| C9JL85 | 1293.383789 | 1319.806763 | 2624.550781 | 0 | 0 | 0 | 0 | 0 | 0 | 0 |
| C9JPG5 | 3051.821045 | 1487.762695 | 1779.770996 | 0 | 0 | 4715.636719 | 0 | 0 | 0 | 0 |
| C9JV77 | 613139.8125 | 310393.9375 | 448569.5313 | 942073.0625 | 651397.8125 | 679765.5625 | 404389.6563 | 1027142.313 | 563380.375 | 1126295.375 |
| C9JWQ3 | 350.6541138 | 935.8135376 | 0 | 1456.851807 | 1639.669678 | 0 | 40022.39453 | 0 | 2264.657715 | 3270.221924 |
| C9JXI5 | 105686.1641 | 79270.57031 | 99887.40625 | 74834.90625 | 110678.6641 | 10156.04688 | 150987.4844 | 29689.35156 | 55557.0625 | 284807.4063 |
| C9JYY6 | 9206.586914 | 13303.77734 | 20010.16016 | 16903.82422 | 10068.94043 | 5092.851074 | 16982.40039 | 10547.89355 | 7362.664063 | 2529.336914 |
| D3YTG3 | 12166.72949 | 12859.76563 | 17563.89844 | 12001.00488 | 6519.306641 | 8052.575195 | 28110.23438 | 12279.90234 | 11641.50391 | 9707.618164 |
| D6RA26 | 27985.83203 | 14097.43848 | 23851.55664 | 126207.25 | 28739.30469 | 189086.5938 | 0 | 181428.5781 | 23078.55273 | 113442.7734 |
| D6RAR4 | 1515.695801 | 3100.493652 | 2009.110718 | 1941.305542 | 0 | 0 | 0 | 0 | 0 | 0 |
| D6RBV2 | 19771.75586 | 15156.50391 | 19216.41016 | 17181.82031 | 11397.8916 | 16613.64453 | 33747.35938 | 15967.82129 | 13221.99023 | 23846.05664 |
| D6RD17 | 11336.41406 | 18528.92969 | 13991.70117 | 8944.545898 | 43714.92578 | 0 | 0 | 29601.11523 | 19374.80859 | 38521.96875 |
| D6RD58 | 14437.50879 | 22025.96094 | 25896.72461 | 14459.49121 | 0 | 29880.18945 | 68181.42969 | 19922.50586 | 23125.2168 | 27022.75195 |
| D6RE82 | 139501.0313 | 1538.240967 | 1740.128174 | 17417.9668 | 3571.266846 | 126856.75 | 3638222.5 | 9887.325195 | 95187.375 | 5694.633789 |
| D6RF35 | 1062213.625 | 965571.625 | 1104153 | 1465084.25 | 1615793.75 | 2121372.25 | 1561060.75 | 3488697.5 | 1910942.375 | 3043181 |
| D6RF86 | 3500.8125 | 1483.310791 | 933.8624268 | 2988.898926 | 2181.267578 | 5371.429199 | 0 | 0 | 0 | 6971.944824 |
| D6RGG3 | 0 | 534.8768921 | 1533.636353 | 0 | 1186.179443 | 0 | 0 | 0 | 0 | 1373.354126 |
| E5RHG6 | 0 | 2003.952271 | 1101.044678 | 2694.623535 | 0 | 0 | 0 | 0 | 0 | 600.3109131 |
| E7EMB3 | 1407.422852 | 4318.562012 | 2288.33667 | 0 | 4494.314453 | 0 | 0 | 0 | 908.6021118 | 3250.939209 |
| E7EMS2 | 70046.25781 | 29966.77734 | 30564.46289 | 30487.40625 | 24908.01367 | 31639.28906 | 129405.9766 | 19674.19336 | 16191.73633 | 51517.14453 |
| E7END6 | 4880.233398 | 4537.91748 | 5178.34375 | 6667.409668 | 6568.810547 | 7437.396973 | 0 | 7219.327637 | 2879.740967 | 0 |
| E7EQB2 | 3887.050781 | 5266.888184 | 3380.245361 | 2597.453613 | 26672.12891 | 11564.15723 | 19387.67383 | 18532.83789 | 17974.32422 | 32620.78516 |
| E7EQR8 | 5199.723633 | 18014.70117 | 28695.3457 | 7986.378418 | 0 | 0 | 0 | 0 | 6774.962891 | 14885.1416 |
| E7ERL0 | 1384.039551 | 2017.493042 | 0 | 4109.180664 | 0 | 0 | 0 | 0 | 0 | 0 |
| E7ES19 | 8502.701172 | 7139.473145 | 12157.35352 | 25780.14844 | 18137.92188 | 55321.69531 | 88043.34375 | 54522.85938 | 13665.32715 | 37708.69922 |
| E7ESB3 | 222.793045 | 1253.860352 | 277.5107117 | 0 | 0 | 2857.178467 | 0 | 5085.938965 | 2205.560303 | 0 |
| E7EU04 | 975.9571533 | 555.2311401 | 0 | 823.3621826 | 0 | 0 | 0 | 0 | 0 | 0 |
| E7EUF1 | 246422.0469 | 249369.0469 | 270116.375 | 209983.375 | 116944.0156 | 229657.3438 | 401155.3438 | 157168.3125 | 150398.2656 | 264178.7188 |
| E7EX29 | 20780.3125 | 19342.81641 | 9962.121094 | 16751.92578 | 11270.52637 | 11360.3457 | 31572.72266 | 5287.637207 | 8265.960938 | 13672.5957 |
| E9PC84 | 0 | 800.6438599 | 1664.919922 | 0 | 2593.646973 | 0 | 0 | 0 | 0 | 0 |
| E9PEK4 | 6531.313477 | 5165.86377 | 6141.033691 | 11961.81641 | 6874.002441 | 3958.817383 | 0 | 8866.459961 | 6852.84668 | 8009.106934 |
| E9PF17 | 5611.887695 | 11273.04688 | 9886.552734 | 1145.968262 | 8459.526367 | 482.2550964 | 8786.103516 | 0 | 1037.310425 | 3212.547852 |
| E9PG40 | 14642.4082 | 21572.34961 | 45475.33594 | 15544.46094 | 20821.40039 | 17684.41016 | 0 | 20770.3125 | 19529.09961 | 9796.470703 |
| E9PGC5 | 798.7896729 | 1834.521118 | 3766.604736 | 3018.317871 | 0 | 0 | 0 | 0 | 1524.516846 | 0 |
| E9PGN7 | 210905.9844 | 200702.25 | 211900.7031 | 126993.0703 | 181824.375 | 97020.50781 | 280589.4688 | 142464.9531 | 253206.6563 | 207828.125 |
| E9PHK0 | 41517.63672 | 22107.79297 | 36201.94141 | 25204.11133 | 35259.42188 | 39995.76953 | 26966.99609 | 27324.72266 | 10758.08789 | 55045.375 |
| E9PIF4 | 4320.451172 | 3639.432861 | 6505.894043 | 8922.276367 | 23268.09375 | 0 | 0 | 0 | 5235.181152 | 0 |
| E9PIK5 | 1976.806763 | 3070.219727 | 1654.731689 | 3917.511475 | 0 | 0 | 0 | 0 | 0 | 809.6175537 |
| E9PK25 | 5974.017578 | 6750.688965 | 0 | 4923.823242 | 0 | 7105.069336 | 12580.12207 | 0 | 0 | 5250.258301 |
| E9PK47 | 0 | 1154.646729 | 0 | 2815.078857 | 985.6934814 | 0 | 0 | 0 | 0 | 0 |
| E9PKC6 | 11763.14551 | 8389.614258 | 0 | 14296.64551 | 0 | 14147.91504 | 296880.5938 | 19161.69531 | 8702.834961 | 0 |
| E9PKE3 | 3808.23291 | 4820.554688 | 3924.437012 | 11533.02832 | 7879.352051 | 4236.804688 | 44865.43359 | 2655.627686 | 3725.915039 | 5350.881836 |
| E9PN95 | 9222.499023 | 9286.942383 | 9229.639648 | 9622.9375 | 4144.148438 | 31862.80078 | 0 | 27412.92188 | 29558.51172 | 14040.68164 |
| E9PPZ9 | 10715.14258 | 12626.10352 | 23752.43555 | 25183.84375 | 16446.13281 | 2911.152588 | 25719.98633 | 94164.375 | 41221.125 | 35133.82813 |
| E9PRU1 | 20634.60352 | 12534.56543 | 19830.00586 | 14327.59473 | 15530.55078 | 20653.35938 | 31008.11133 | 21547.09766 | 17147.79102 | 9667.464844 |
| F5GWQ8 | 1333.993042 | 3179.866943 | 3476.480469 | 0 | 0 | 0 | 0 | 7288.038086 | 0 | 0 |
| F5GXJ9 | 3642.246826 | 2712.830322 | 2516.578613 | 2732.680908 | 1549.237305 | 0 | 0 | 0 | 1136.278564 | 3785.705566 |
| F5GY80 | 6902.023438 | 8927.838867 | 8301.543945 | 12117.2627 | 6560.751465 | 7811.585938 | 14713.89063 | 7389.319336 | 8999.210938 | 2951.091309 |
| F5GZI0 | 1350.687378 | 2733.379883 | 2302.02832 | 0 | 0 | 0 | 0 | 0 | 1134.761597 | 0 |
| F5H1S8 | 1363.559937 | 754.8626709 | 730.8201904 | 0 | 0 | 0 | 0 | 0 | 0 | 0 |
| F5H283 | 13692.82422 | 5276.210938 | 7536.158203 | 17904.0957 | 5277.185547 | 22263.75781 | 52336.01563 | 13443.3623 | 8036.931152 | 1336.892822 |
| F5H5D3 | 593.7672729 | 779.802124 | 226.4010315 | 959.6188354 | 0 | 0 | 0 | 0 | 0 | 0 |
| F6S8M0 | 23412.54492 | 23877.35742 | 27433.79883 | 13582.22363 | 8476.026367 | 16820.08008 | 0 | 8348.629883 | 3771.319336 | 9228.459961 |
| F6SYF8 | 29485.61523 | 9228.513672 | 15533.97852 | 34111.70703 | 29332.98828 | 13210.50293 | 0 | 10061.26367 | 46929.53516 | 3429.779785 |
| F6VDH7 | 5502.299316 | 2113.138428 | 5052.861328 | 1502.887085 | 2313.034668 | 546.3602905 | 0 | 794.7932739 | 1497.98877 | 594.2340698 |
| F6WFR7 | 7917.667969 | 11891.84277 | 18448.92188 | 13688.41895 | 0 | 0 | 0 | 0 | 0 | 0 |
| F8VVB6 | 17344.82227 | 11315.10645 | 26726.92383 | 11331.25977 | 5948.642578 | 31212.13477 | 0 | 26909.68945 | 15849.10352 | 30740.35938 |
| F8W1S1 | 0 | 704.7634277 | 0 | 0 | 0 | 7311.634277 | 0 | 2382.544189 | 13430.72461 | 4778.849121 |
| F8W785 | 4227.587891 | 2863.546631 | 4254.183594 | 2016.618896 | 1058.759888 | 0 | 0 | 0 | 0 | 0 |
| F8W8W4 | 2933.946289 | 1746.90979 | 3464.453369 | 1480.213501 | 790.8561401 | 0 | 0 | 0 | 13970.5918 | 1766.414795 |
| F8WF14 | 6348.189941 | 13606.93164 | 6199.063477 | 4628.313477 | 5183.939453 | 6917.371582 | 0 | 18617.86328 | 12364.27832 | 0 |
| G3V1C5 | 12311.22363 | 4529.214355 | 0 | 0 | 0 | 6326.254395 | 0 | 13205.39453 | 0 | 3339.556641 |
| G3V2W1 | 2493.729736 | 1864.929688 | 0 | 0 | 3313.885742 | 0 | 0 | 0 | 4503.096191 | 0 |
| G3V3X5 | 133822.1406 | 46419.05859 | 127478.3906 | 68151 | 60570.73047 | 199340.125 | 209017.0781 | 139176.1094 | 61632.64844 | 53187.47266 |
| G3V4U0 | 11171.30371 | 9270.62793 | 9169.400391 | 9671.921875 | 9940.245117 | 12888.89453 | 0 | 0 | 6788.580566 | 14871.375 |
| G3XAK1 | 4354.756836 | 5606.243652 | 3767.983887 | 1938.416748 | 5696.869629 | 5935.728516 | 28061.02148 | 5973.11084 | 6271.035156 | 4220.173828 |
| G5E9G7 | 0 | 7047.447754 | 6897.939453 | 4449.341309 | 0 | 3479.588867 | 0 | 0 | 0 | 0 |
| H0Y6T7 | 1749.292603 | 2517.995361 | 2637.572266 | 0 | 550.4697266 | 0 | 0 | 0 | 0 | 0 |
| H0Y9C7 | 6928.482422 | 7507.32373 | 9434.463867 | 11153.91895 | 6589.324707 | 6310.10791 | 0 | 2527.778076 | 7082.33252 | 14670.93945 |
| H0YAC1 | 8651.697266 | 16772.11523 | 10924.18359 | 6281.476074 | 15874.08789 | 13843.91797 | 74966.78906 | 35642.14844 | 31985.04688 | 9067.024414 |
| H0YB13 | 3835.632568 | 4507.001465 | 5807.792969 | 519.4377441 | 0 | 0 | 0 | 0 | 0 | 5517.806641 |
| H0YF95 | 9352.298828 | 8890.803711 | 15093.20508 | 11434.16992 | 6201 | 6050.809082 | 0 | 9713.870117 | 6222.679688 | 0 |
| H0YH81 | 915.9122925 | 203.6334686 | 480.9631958 | 2183.256592 | 0 | 0 | 0 | 0 | 0 | 0 |
| H3BLU2 | 18442.6875 | 12654.95117 | 16054.55371 | 20405.69531 | 6970.442871 | 21369.66406 | 4194.35791 | 21206.96484 | 9925.949219 | 14125.68359 |
| H3BP20 | 14954.20801 | 4475.592285 | 13222.18945 | 8230.856445 | 8827.416992 | 14700.35547 | 32375.01758 | 8943.298828 | 9156.882813 | 8211.955078 |
| H7BY57 | 3433.568359 | 4405.114746 | 7596.78125 | 4332.72998 | 5737.214355 | 0 | 0 | 0 | 2771.021973 | 0 |
| H7BY64 | 19281.87891 | 11748.78809 | 5850.084961 | 9341.460938 | 811.835022 | 50101.11719 | 22123.80859 | 36743.17969 | 35957.14844 | 0 |
| H7C2K7 | 1067.396362 | 2472.567871 | 2777.071777 | 2769.660645 | 2088.018311 | 3587.704102 | 0 | 4534.879395 | 0 | 0 |
| H7C2R7 | 16393.71484 | 24061.93164 | 406348.6563 | 26636.22852 | 684937.5 | 834777.625 | 704970.5 | 1411430.75 | 0 | 22905.3457 |
| I3L1J2 | 8441.310547 | 10212.59766 | 6578.756836 | 10280.85547 | 7595.10498 | 9832.25 | 0 | 9104.675781 | 13255.7793 | 11422.75977 |
| I3L2R6 | 3011.052979 | 2425.342285 | 4556.070313 | 2384.685791 | 15882.77441 | 4989.293945 | 0 | 1249.156738 | 829.8903809 | 3273.806641 |
| I3L3J8 | 1929.437988 | 822.1630859 | 2382.653564 | 2061.080322 | 0 | 0 | 0 | 0 | 5355.812012 | 0 |
| J3KNP4 | 18622.79297 | 20553.36719 | 18413.67188 | 19299.22461 | 12730.51074 | 17608.76953 | 26159.13477 | 21794.51172 | 15081.95996 | 9044.470703 |
| J3KPS3 | 5739.293945 | 4305.82373 | 5155.809082 | 5575.231934 | 2762.234131 | 4774.239746 | 14373.94629 | 2992.702881 | 2090.429199 | 0 |
| J3KQ66 | 15356.73633 | 6101.098145 | 10156.26563 | 6926.212891 | 8037.136719 | 22660.31445 | 20300.06836 | 7397.540527 | 8335.477539 | 9765.851563 |
| J3KSN0 | 3530.134277 | 9929.313477 | 3397.931396 | 5001.994141 | 1579.794922 | 17757.76367 | 0 | 10476.08105 | 11678.33203 | 8111.288574 |
| K7EIS2 | 37017.38281 | 13084.03516 | 90042.78125 | 12372.61914 | 15226.49512 | 79683.26563 | 78104.42188 | 44324.44141 | 18320.87109 | 4242.309082 |
| K7ELL7 | 11359.61914 | 6409.82959 | 12495.40234 | 7957.476563 | 4768.622559 | 6032.694336 | 0 | 8604.75293 | 3674.856934 | 3620.705078 |
| K7EN15 | 3764.583008 | 3759.197266 | 3057.075195 | 0 | 1632.253784 | 0 | 0 | 922.4164429 | 0 | 0 |
| K7ERG9 | 88889.34375 | 131160.6563 | 121730.9375 | 85477.13281 | 169224.8594 | 409922.9375 | 167138.9219 | 184473.8125 | 303417.8125 | 347179.25 |
| K7ERI9 | 114340.8828 | 6298.985352 | 94924.5 | 763.6751099 | 307909.75 | 198458.1875 | 0 | 620372.875 | 225768.3906 | 675231.625 |
| O00115 | 9609.936523 | 9072.410156 | 10596.92383 | 7138.981445 | 7595.322266 | 24868.52734 | 40171.85547 | 9927.469727 | 5807.975586 | 15629.56348 |
| O00187 | 13636.36523 | 15967.9707 | 0 | 0 | 0 | 32520.77344 | 88278.07031 | 75692.27344 | 24578.62109 | 0 |
| O00292 | 3795.198975 | 717.8217163 | 4411.237305 | 4136.894043 | 6378.869141 | 4535.25 | 10550.68359 | 11325.7832 | 7319.143555 | 2218.45459 |
| O00391 | 21127.66797 | 18958.19141 | 21680.79883 | 15372.17773 | 18349.11328 | 19146.5332 | 56077.35938 | 16431.32227 | 17450.75977 | 18705.89063 |
| O14498 | 20476.85938 | 15861.31152 | 13888.83398 | 18127.81836 | 14411.44531 | 22935.17383 | 4500.438477 | 16583.10352 | 11922.61328 | 29208.88672 |
| O14791 | 2934.571289 | 6499.276855 | 0 | 2152.289795 | 0 | 0 | 22626.17773 | 6886.21875 | 5255.179199 | 0 |
| O15031 | 8628.364258 | 3793.453613 | 8857.950195 | 4905.012695 | 3108.791748 | 3551.89917 | 0 | 0 | 1912.036011 | 0 |
| O15240 | 4376.105469 | 1791.307495 | 4566.601563 | 1253.466309 | 1698.514893 | 4288.75293 | 0 | 5725.375 | 1346.227539 | 0 |
| O15537 | 22805.92188 | 16000.84863 | 34031.24219 | 14129.83691 | 15560.24902 | 28733.30859 | 25637.25977 | 7639.816895 | 11619.45117 | 3348.01001 |
| O43505 | 53390.00781 | 53444.07422 | 96726.26563 | 64730.96875 | 48607.875 | 44877.70703 | 92723.71875 | 55576.57813 | 49054.94141 | 31715.30469 |
| O43707 | 4624.090332 | 3608.05835 | 7729.588867 | 6220.606445 | 8016.884277 | 7268.702148 | 0 | 5642.32666 | 2712.407959 | 28697.97852 |
| O43827 | 11098.85156 | 9817.006836 | 12376.54688 | 22362.1582 | 15016.18359 | 16712.0625 | 5938.75293 | 7741.594238 | 6824.911621 | 41369.51172 |
| O43866 | 17756.00391 | 20338.48828 | 11348.85254 | 6474.790527 | 36449.64063 | 10535.54004 | 0 | 37566.10156 | 25315.32813 | 12399.38086 |
| O60575 | 5728.041504 | 9444.163086 | 5449.929199 | 4513.789551 | 2915.556152 | 6606.056641 | 0 | 2657.255859 | 1524.563477 | 3393.580566 |
| O60814 | 2463.816406 | 4394.897949 | 14277.30566 | 2954.649658 | 5992.525879 | 5843.564941 | 38448.95703 | 4346.247559 | 30692.67969 | 8396.398438 |
| O60938 | 40019.49609 | 46399.9375 | 47616.15625 | 84495.11719 | 32392.04688 | 50701.28906 | 103372.5859 | 57050.22656 | 19064.14648 | 72202.8125 |
| O75093 | 14637.09473 | 32214.96875 | 26960.51758 | 18913.92969 | 13152.55176 | 16039.88184 | 60102.17188 | 16374.57715 | 12269.66309 | 11758.06445 |
| O75326 | 12230.67871 | 24640.91406 | 38627.96484 | 13859.47656 | 9117.712891 | 10836.8457 | 25742.12695 | 1382.153564 | 6798.064453 | 23166.30859 |
| O75368 | 4729.745117 | 3270.797852 | 2523.798828 | 5380.910156 | 1531.944824 | 8749.917969 | 0 | 6140.38623 | 2587.884277 | 4258.880859 |
| O75636 | 2089.670898 | 8561.129883 | 2145.302979 | 857.1896362 | 3858.600342 | 0 | 0 | 9435.614258 | 7588.199219 | 0 |
| O75752 | 467.5783691 | 799.3792725 | 0 | 6966.990234 | 0 | 0 | 0 | 0 | 0 | 0 |
| O75882 | 14371.06445 | 21960.45703 | 14679.74805 | 14369.78027 | 9264.114258 | 7627.509766 | 13216.37207 | 10038.84766 | 11825.63672 | 12170.70801 |
| O94919 | 2836.975342 | 3872.131104 | 3583.749512 | 4597.209961 | 3741.023682 | 5908.245605 | 0 | 6599.546387 | 5486.100098 | 5300.719238 |
| O94985 | 365141.4688 | 302444.875 | 345527.9375 | 333858.1875 | 204554.75 | 326567.1875 | 68032.35938 | 297923.75 | 217656.3281 | 354308.25 |
| O95274 | 194616.5156 | 24061.90039 | 406348.6563 | 26636.27539 | 684942.4375 | 834777.625 | 704990 | 1413083.375 | 0 | 22905.87305 |
| O95428 | 7857.564941 | 5427.496094 | 10497.39746 | 4888.810547 | 4905.222168 | 10728.99609 | 24454.83594 | 10715.33105 | 5105.378418 | 0 |
| O95445 | 5818.677734 | 6882.613281 | 4333.586914 | 4239.864258 | 8551.681641 | 3977.936035 | 0 | 3500.394531 | 2441.185547 | 0 |
| O95497 | 0 | 3948.230225 | 2536.233398 | 5002.040039 | 2474.036621 | 0 | 0 | 3022.791016 | 0 | 2387.398926 |
| P00338 | 33105.35938 | 23111.60742 | 25075.77734 | 56802.33984 | 22088.73047 | 25104.61914 | 57521.89844 | 18467.14063 | 22298.64453 | 36458.40234 |
| P00352 | 6664.861816 | 9770.267578 | 4740.188965 | 21064.82422 | 4626.775879 | 10356.71094 | 44926.97656 | 5833.314941 | 1651.172241 | 13407.68652 |
| P00390 | 4726.189941 | 5276.318848 | 6820.572754 | 4552.874023 | 0 | 0 | 0 | 3780.604248 | 2299.493408 | 0 |
| P00441 | 10332.9082 | 7318.063965 | 12180.10449 | 17851.39648 | 5088.583008 | 16176.37207 | 0 | 5284.924805 | 6507.210449 | 20625.37305 |
| P00450 | 932860.125 | 815927.1875 | 1250726.375 | 999691.8125 | 790324.5 | 1137469.625 | 447801.9375 | 1736328.125 | 971446.4375 | 1048755.75 |
| P00491 | 0 | 2743.085205 | 5412.643555 | 3506.763184 | 0 | 0 | 0 | 0 | 2026.650879 | 0 |
| P00558 | 5556.244141 | 4299.818848 | 3232.349121 | 8930.539063 | 4809.557129 | 10947.5957 | 29074.02734 | 5110.000488 | 9078.115234 | 4304.824707 |
| P00568 | 393.1643372 | 0 | 872.8110352 | 777.347168 | 0 | 0 | 3.204241514 | 0 | 0 | 0 |
| P00734 | 169729.0313 | 118844.5 | 160592.4688 | 211854.7813 | 182428.1094 | 247811.0469 | 296001.125 | 411370.375 | 134236.9375 | 198764.1406 |
| P00738 | 6500.214355 | 1881801.875 | 20277.48242 | 14294.62402 | 361712.0938 | 1128032.75 | 3454176 | 177708.3281 | 66977.28125 | 26636.41602 |
| P00740 | 1873.657104 | 3286.184082 | 3163.733154 | 3021.878174 | 2452.761719 | 0 | 0 | 2835.939941 | 3150.842773 | 0 |
| P00742 | 26306.91016 | 16306.44238 | 14352.63574 | 25423.47266 | 24244.26172 | 38277.84766 | 79261.36719 | 72536.5625 | 20741.81055 | 44292.83984 |
| P00747 | 318918.1563 | 282052.0938 | 286727.5313 | 346256.9375 | 497877.4688 | 605118.9375 | 1598131 | 758400.4375 | 525235.5625 | 506984.75 |
| P00748 | 88807.11719 | 85248.42969 | 94178.9375 | 64086.46484 | 83300.79688 | 46790.86719 | 95662.77344 | 129207.7344 | 56609.97656 | 97525.20313 |
| P00915 | 90655.72656 | 56367.53125 | 170142.7969 | 117395.6328 | 34677.96484 | 34280.05469 | 15866.66406 | 33214.55469 | 140410.5 | 119051.0938 |
| P00918 | 11432.96387 | 11265.90137 | 12440.75391 | 11789.62988 | 3591.860107 | 0 | 0 | 0 | 12365.18066 | 11066.73633 |
| P01008 | 582330.5625 | 418652.4375 | 405342.1563 | 878216 | 788496.5625 | 1051049.625 | 1568421.75 | 1587081.625 | 921851.25 | 1254159.375 |
| P01009 | 1294741.5 | 1020614.5 | 1064010.75 | 1284209.875 | 2031802 | 1114791.25 | 3676207.5 | 3616841 | 1677554.25 | 2537350.5 |
| P01011 | 1443116.75 | 666891.375 | 1155869.5 | 1149808.375 | 1048534.688 | 871885.5 | 1077526.625 | 2344465.25 | 1460541.375 | 1172499.125 |
| P01019 | 121685.1797 | 146826.9375 | 162757.4063 | 201273.2813 | 282878.1875 | 138441.4063 | 360113.2813 | 251224.3125 | 189193.8438 | 291760.0938 |
| P01023 | 682280.75 | 1051021.75 | 766765.4375 | 341500.7188 | 626752.375 | 623560.1875 | 650649.9375 | 794033.3125 | 644018.8125 | 287702.5625 |
| P01024 | 508061.5313 | 730092.875 | 585646 | 554643.9375 | 618630.3125 | 660850.9375 | 2136579.5 | 1284643.375 | 1031247.063 | 918247 |
| P01031 | 36661.33203 | 56773.5 | 42312.625 | 41374.07422 | 51637.67969 | 64825.5 | 174959.2969 | 205330.9688 | 98623.28125 | 58087.23828 |
| P01033 | 28746.57227 | 25403.22852 | 23459.89258 | 17504.12695 | 23338.60547 | 19913.16602 | 32229.65039 | 36003.54297 | 13902.61914 | 26254.68945 |
| P01034 | 1053903 | 1218921.625 | 1624265.5 | 808631.5 | 851833.8125 | 2875455 | 4450706 | 1226683.625 | 994281.0625 | 1771902.125 |
| P01042 | 404004.0938 | 269369.625 | 332851.875 | 587204.125 | 433185.7188 | 437692.1563 | 371862.5 | 729977.75 | 632118.8125 | 495524.4688 |
| P01344 | 3796.561279 | 2472.587402 | 2520.571777 | 2416.736572 | 0 | 2670.810303 | 0 | 0 | 0 | 6329.355957 |
| P01599 | 6176.285156 | 14846.46094 | 20006.68945 | 10188.7793 | 7242.65625 | 4816.92334 | 39238.16797 | 23893.14063 | 11862.60742 | 11048.00391 |
| P01602 | 163199.7031 | 209511.8906 | 520225.5 | 219183.7031 | 394012.9063 | 640945.375 | 1304198.125 | 1201344.125 | 511196.375 | 431458.4063 |
| P01619 | 482983 | 510070.9375 | 1009685 | 433308.4063 | 552895.5625 | 904889.375 | 4717879.5 | 2253586.5 | 914546.75 | 1101093.75 |
| P01624 | 381711.875 | 314614.4688 | 552015.5 | 239430.375 | 485079.2813 | 715439.8125 | 2431322.5 | 1927053.125 | 439070.2188 | 590317.375 |
| P01700 | 270937.3438 | 157063.6875 | 242795 | 150059.7813 | 142753.9063 | 125396.3594 | 654581.375 | 326750.4688 | 168880.0313 | 193218.7188 |
| P01701 | 45455.73828 | 57549.69922 | 86186.52344 | 35472.5 | 79776.77344 | 56785.14844 | 260467.3438 | 51170.64453 | 62391.04688 | 100837.4219 |
| P01704 | 38633.25 | 12415.12402 | 5033.214844 | 33263.84766 | 14767.41113 | 32984.67188 | 0 | 17822.33008 | 11388.30371 | 55109.07422 |
| P01705 | 9525.546875 | 18454.37305 | 13308.5752 | 4405.646973 | 10501.75098 | 39668.8125 | 55408.45313 | 17960.57813 | 0 | 0 |
| P01706 | 5288.134277 | 57777.98438 | 58089.50391 | 10479.13574 | 9571.136719 | 88655.75781 | 372454.4063 | 28175.46484 | 91409.45313 | 106718.1797 |
| P01709 | 17466.91406 | 25320.19141 | 29239.20898 | 23054.65625 | 26703.47461 | 33653.61328 | 113033.4063 | 60108.42969 | 31307.77734 | 47096.14844 |
| P01714 | 80096.23438 | 22596.68164 | 17101.54297 | 62255.875 | 48788.86719 | 38599.95313 | 127748.5938 | 85956.91406 | 41132.77344 | 108559.1875 |
| P01742 | 8020.952637 | 8402.198242 | 11985.7168 | 0 | 7751.461914 | 0 | 0 | 0 | 0 | 4197.80957 |
| P01766 | 13935.44727 | 16517.86133 | 83756.95313 | 10594.11621 | 12573.00586 | 8958.586914 | 40854.07422 | 16316.23633 | 2279.0625 | 26724.47461 |
| P01780 | 64210.65234 | 68356.49219 | 153687.3594 | 71991.125 | 97150.53906 | 83818.4375 | 310136.5 | 116747.0469 | 79861.42188 | 204996.2813 |
| P01834 | 1737098.125 | 2720684.5 | 4492363.5 | 1673551.875 | 4390138 | 2576411.25 | 5982332 | 4627560 | 3419057.25 | 4400858 |
| P02042 | 90389.09375 | 33743.22266 | 68705.60938 | 71170.40625 | 27621.44531 | 29254.64063 | 84800.39063 | 31290.51172 | 79824.09375 | 22769.84766 |
| P02452 | 21987.57617 | 4868.787109 | 6982.119629 | 21420.74219 | 13014.97656 | 16079.64941 | 0 | 0 | 6322.487305 | 4115.423828 |
| P02458 | 1064.333252 | 2211.401855 | 3603.070557 | 1132.258301 | 0 | 0 | 0 | 0 | 0 | 0 |
| P02461 | 5161.35498 | 1743.66394 | 0 | 4515.125977 | 2332.792969 | 3844.738037 | 0 | 0 | 3682.662598 | 1948.978027 |
| P02533 | 3810.503906 | 6441.355469 | 2109.137451 | 4356.012695 | 4806.084961 | 5387.60791 | 55763.22266 | 35889.10938 | 0 | 8778.441406 |
| P02538 | 0 | 1771.230225 | 0 | 4519.114258 | 0 | 0 | 33771.38672 | 8479.099609 | 4359.400879 | 0 |
| P02647 | 1236374.25 | 1652729.5 | 1026862.438 | 1220026.75 | 2578171.5 | 2550308.75 | 856387.9375 | 3436958 | 1315705.375 | 2102341 |
| P02649 | 627451.375 | 620860.375 | 822833.9375 | 369159.0313 | 940796.9375 | 574381.25 | 1245240.875 | 2068084 | 607032.4375 | 291874.75 |
| P02652 | 255112.0625 | 270040.6563 | 206896.0938 | 252243.2656 | 385778.7813 | 469939.0313 | 283210.8125 | 852456.875 | 256296.875 | 440944.6875 |
| P02671 | 169937.7813 | 211649.8438 | 101342.1953 | 45363.22656 | 152119.1719 | 215707.1719 | 767895.375 | 869368.375 | 295208.0625 | 18746.94922 |
| P02675 | 112615.9766 | 186485.9531 | 114691.2969 | 48053.80078 | 124803.5859 | 94532.5 | 561122.875 | 980692.0625 | 215322.5625 | 4081.342285 |
| P02741 | 0 | 1439.89917 | 6453.854004 | 1388.141968 | 0 | 0 | 0 | 25950.62891 | 39178.45313 | 3011.880859 |
| P02743 | 25725.03125 | 49798.73438 | 16821.25 | 28880.82617 | 30224.63086 | 35171.71875 | 117613.625 | 314719.5938 | 88521.32813 | 47781.62109 |
| P02745 | 16847.08984 | 17327.26758 | 15165.55957 | 13547.74512 | 40342.10547 | 40288.14453 | 79977.89063 | 58071.41797 | 32736.39258 | 0 |
| P02747 | 53957.58984 | 53797.34375 | 41984.89844 | 34871.78125 | 44502.1875 | 75469.67188 | 157421.8594 | 91393.80469 | 48028.98438 | 28859.78516 |
| P02748 | 114750.3594 | 118186.2109 | 107539.7656 | 135832.9375 | 148425.8281 | 193924.0156 | 76259 | 410109.8125 | 237659.0938 | 148390.375 |
| P02749 | 831449.1875 | 811267.0625 | 579278.625 | 1035108.688 | 771915.75 | 1950030 | 6539613 | 3273205 | 1611737.5 | 1216536 |
| P02750 | 466295.1875 | 352349.625 | 368877.3125 | 405163.5313 | 549882.5 | 1326494.5 | 383508.9063 | 2508313.75 | 887129.5 | 844904.25 |
| P02751 | 154454.4688 | 66030.25781 | 43037.94141 | 85508.96094 | 168287.5313 | 42149.43359 | 62818.59766 | 85999.71875 | 125051.9844 | 12306.54492 |
| P02753 | 159250.9688 | 217941.2969 | 133203.6719 | 114802.5 | 80285.07031 | 324937.8438 | 697853.375 | 278492.9063 | 310913.4375 | 151960.5313 |
| P02760 | 279044.8125 | 372606.1875 | 152996.8438 | 160079.25 | 167248.4375 | 491414.7813 | 625229.125 | 692336 | 966248.1875 | 300046.9688 |
| P02763 | 1340355.875 | 875870.375 | 1269757.75 | 1549628.25 | 1288644 | 2985398 | 1351381.125 | 7812651 | 3083540.5 | 2640762.5 |
| P02766 | 3317428.5 | 2403708 | 2294406 | 2195164.25 | 2473461 | 547105.3125 | 2083621.875 | 965547.75 | 2292380.5 | 2603799.75 |
| P02768 | 118713104 | 57368628 | 88645144 | 154541232 | 146930560 | 104492664 | 169038992 | 212924992 | 134882480 | 275963424 |
| P02775 | 0 | 12011.39258 | 14674.09375 | 3400.102295 | 14743.05566 | 9283.811523 | 50600.39063 | 111974.6719 | 41966.23438 | 6616.704102 |
| P02787 | 9832409 | 8406888 | 13134078 | 9918862 | 13292647 | 9545046 | 38176164 | 12278333 | 9444568 | 16497034 |
| P02790 | 440814.1875 | 570167.625 | 370469.2188 | 597867.1875 | 802231.4375 | 1403522.75 | 899292.75 | 901858.625 | 400473.4688 | 1250239.5 |
| P03950 | 23139.24023 | 19100.40039 | 12910.89551 | 13954.02734 | 6487.11377 | 40223.46484 | 127955.5469 | 19599.72852 | 20156.83789 | 19619.78125 |
| P03951 | 6032.337891 | 3672.595459 | 2802.907227 | 3323.034668 | 4163.794434 | 6751.650391 | 0 | 21268.70508 | 9942.793945 | 9080.806641 |
| P03973 | 2447.584717 | 2395.447021 | 0 | 3065.341064 | 0 | 6226.019043 | 0 | 0 | 3176.226807 | 5729.076172 |
| P04003 | 17564.75 | 18743.59375 | 4856.395996 | 10883.39844 | 11717.99609 | 8560.725586 | 49991.05078 | 5004.176758 | 10085.38965 | 33288.35938 |
| P04004 | 365390.75 | 293764.75 | 337385.6875 | 563613.3125 | 670665.5625 | 1124539.75 | 2079758.625 | 1273254.5 | 493464.5938 | 146932.8438 |
| P04040 | 6118.166504 | 2030.936646 | 12877.38184 | 11025.0166 | 2464.192871 | 0 | 0 | 11068.16895 | 10496.86914 | 0 |
| P04066 | 10043.04883 | 9133.753906 | 8859.824219 | 7041.339355 | 7136.779297 | 0 | 0 | 4528.794922 | 3951.151611 | 0 |
| P04114 | 27961.76758 | 46294.58594 | 17057.74609 | 3576.973633 | 25221.375 | 4158.027344 | 26441.26563 | 32089.87109 | 20009.09375 | 2418.677734 |
| P04196 | 188789.0781 | 354091.5938 | 346828.3438 | 442289.9063 | 422357.3438 | 327566.25 | 509783.0625 | 257575.8281 | 279071.5625 | 658154.75 |
| P04211 | 2420.662354 | 0 | 0 | 4423.011719 | 0 | 27604.46875 | 93111.25781 | 15857.53223 | 3671.584229 | 5503.187012 |
| P04217 | 731393.5 | 707181.75 | 640229.4375 | 802714.875 | 841601.75 | 1009060.313 | 648661.3125 | 1571237.625 | 825779.75 | 1260530.25 |
| P04259 | 24991.73633 | 74784.375 | 19112.47461 | 29394.65039 | 79435.11719 | 10753.00098 | 190890.7813 | 199606.7188 | 30935.60352 | 17788.25586 |
| P04264 | 67576.17188 | 206455.9688 | 105263.8047 | 72760.50781 | 280204.3438 | 25227.41211 | 318729.8438 | 1135201.375 | 65611.71875 | 42499.80469 |
| P04275 | 3664.29541 | 1799.828125 | 2026.955078 | 2996.424561 | 1443.850098 | 0 | 0 | 5275.15918 | 2073.27124 | 0 |
| P04406 | 36143.32031 | 19328.17773 | 34927.39844 | 33287.17969 | 16127.64551 | 10425.6709 | 284253.375 | 35015.05469 | 32082.07031 | 10438.2793 |
| P04430 | 10160.70313 | 20941.96289 | 7035.95752 | 0 | 0 | 12458.13965 | 0 | 17858.06055 | 0 | 0 |
| P04792 | 4745.066895 | 5703.928711 | 5557.227539 | 9716.791992 | 5478.523438 | 6662.135742 | 67809.64844 | 11922.49121 | 0 | 0 |
| P05060 | 14666.20508 | 5177.835449 | 11035.29004 | 7641.524414 | 0 | 4285.449707 | 0 | 0 | 2563.521729 | 0 |
| P05121 | 2237.747314 | 2093.907227 | 780.5795288 | 1445.731689 | 5651.691406 | 0 | 0 | 0 | 0 | 0 |
| P05154 | 56408.37891 | 69304.58594 | 73234.60938 | 76074.53906 | 53772.95313 | 72801.09375 | 156582.0781 | 137561.1875 | 59900.10938 | 67990.5625 |
| P05160 | 2814.572266 | 3280.275391 | 2058.330811 | 989.8619995 | 39831.65625 | 8074.498535 | 4737.717285 | 5558.554688 | 2855.79541 | 0 |
| P05408 | 65060.49219 | 79988.67969 | 95840.89063 | 64755.50781 | 38306.32422 | 132554.0156 | 11211.06836 | 155247.625 | 36447.33203 | 163657.4688 |
| P05413 | 4429.56543 | 2181.376953 | 2212.219971 | 0 | 4663.643066 | 9233.646484 | 0 | 0 | 9058.34082 | 8961.140625 |
| P05543 | 33070.71094 | 21832.63281 | 38040.33984 | 38088.41406 | 34841.31641 | 23633.89844 | 14380.73145 | 34344.26172 | 34647.10938 | 76271.10938 |
| P05546 | 183665.0625 | 153527.875 | 177124.0313 | 223991.9375 | 337621.9688 | 325636.8438 | 784174.125 | 613105.125 | 351947.9375 | 154788 |
| P05976 | 0 | 191.2974091 | 0 | 0 | 0 | 9933.663086 | 0 | 2538.592529 | 457.9432373 | 0 |
| P06310 | 31744.91016 | 85398.53906 | 64186.93359 | 71313.07813 | 63915.125 | 277505.7188 | 29024.65234 | 289299.375 | 84478.35156 | 130692.4141 |
| P06312 | 107644.5156 | 127836.4531 | 135318.375 | 83346.16406 | 65826.48438 | 142260.1563 | 162761.4063 | 221552.1406 | 78309.50781 | 234222.5938 |
| P06396 | 459814.0938 | 302949.8125 | 429539 | 324416.6875 | 543318.875 | 376850.0313 | 797243.875 | 549440.4375 | 441833.875 | 421011.6875 |
| P06681 | 13890.28809 | 25510.80273 | 17040.68359 | 26992.45313 | 15960.63184 | 17069.42773 | 42616.32031 | 27349.01953 | 26419.8457 | 28366.87109 |
| P06702 | 0 | 2146.272217 | 1602.608154 | 0 | 2115.576172 | 0 | 0 | 0 | 4800.946289 | 0 |
| P06703 | 47419.17188 | 25254.90039 | 6655.625488 | 69151.49219 | 23147.77539 | 21970.36914 | 358866.9688 | 26772.26953 | 31513.65625 | 29740.3418 |
| P06727 | 1079073.75 | 716268.625 | 502043.8125 | 824329.1875 | 945055.0625 | 2647355.75 | 700534.375 | 3364427.5 | 1382679.25 | 406173.5625 |
| P06733 | 38433.96484 | 52716.35938 | 31823.01563 | 124118.8125 | 21329.88281 | 30483.08789 | 185853.2188 | 34571.05859 | 51958.80469 | 54696.92188 |
| P07195 | 6493.022461 | 7110.166992 | 6369.558105 | 11763.53711 | 5608.146484 | 7819.918945 | 91849.89844 | 8124.561523 | 6936.958496 | 7034.89502 |
| P07315 | 3378.632324 | 2701.050781 | 10058.83203 | 3742.130371 | 0 | 7707.39502 | 0 | 14225.43652 | 606.9308472 | 18264.88867 |
| P07357 | 15581.78418 | 19740.23242 | 17899.74023 | 19741.06836 | 20247.62695 | 36557.95313 | 90393.21875 | 71160.92188 | 27941.94531 | 24709.57031 |
| P07360 | 32538.29688 | 39733.30469 | 27214.06055 | 37746.25781 | 33898.14063 | 50400.83594 | 321547.0625 | 25300.86719 | 67436.08594 | 29732.73438 |
| P07384 | 2145.946045 | 906.2944946 | 0 | 1270.233887 | 0 | 0 | 0 | 0 | 0 | 0 |
| P07437 | 0 | 5097.330566 | 0 | 4594.336914 | 276992.0313 | 0 | 0 | 1096.103027 | 0 | 0 |
| P07451 | 12915.29297 | 6276.075684 | 13128.87598 | 31723.0293 | 0 | 0 | 69068.52344 | 0 | 14802.76758 | 10316.15723 |
| P07585 | 40208.20313 | 33030.30469 | 38782.75391 | 54122.35938 | 54922.87109 | 61013.28516 | 136828.7813 | 79474.01563 | 16186.64355 | 48258.70703 |
| P07686 | 15622.50488 | 10163.13086 | 17055.57617 | 9969.171875 | 7023.92334 | 12780.55469 | 19747.01367 | 9141.563477 | 7339.437988 | 8917.504883 |
| P07711 | 22537.57031 | 21405.78516 | 30552.20508 | 20061.52539 | 12340.36914 | 22925.56641 | 27971.47461 | 13530.27832 | 12378.04492 | 19341.9668 |
| P07737 | 5729.529785 | 10354.8125 | 4810.921875 | 5113.706055 | 7054.184082 | 56291.50781 | 0 | 230608.6094 | 7901.244141 | 7510.626953 |
| P07738 | 3474.662842 | 1676.484863 | 6837.976563 | 3508.014404 | 0 | 0 | 0 | 0 | 2234.19043 | 0 |
| P07858 | 10346.62305 | 7762.671875 | 10815.91797 | 8123.505371 | 9876.382813 | 12135.57129 | 41950.95313 | 0 | 9984.558594 | 7729.03125 |
| P07900 | 1655.373901 | 1712.636475 | 2164.811279 | 2191.488525 | 717.0977173 | 0 | 0 | 0 | 0 | 0 |
| P07996 | 3804.061279 | 2352.264648 | 2171.585938 | 2046.575562 | 8726.585938 | 4076.82373 | 0 | 0 | 3271.27417 | 7785.807129 |
| P07998 | 257036.9063 | 180366.2031 | 148059.4531 | 133836.3438 | 79223.97656 | 220747.9688 | 127370.9766 | 88968.58594 | 90891.52344 | 215823.8281 |
| P08185 | 92896.78125 | 49648.28516 | 68369.75781 | 85663.875 | 66387.41406 | 73054.125 | 115530.7813 | 120735.9219 | 85041.53125 | 190367.5625 |
| P08253 | 29720.26758 | 28415.49414 | 24396.98438 | 32683.66016 | 33304.16016 | 45588.12891 | 20854.89844 | 16992.07617 | 23997.55664 | 36481.76953 |
| P08294 | 62835.05859 | 31661.10547 | 75720.00781 | 62949.71875 | 38857.97656 | 66848.92188 | 92014.59375 | 93918.59375 | 36132.68359 | 111986.3438 |
| P08493 | 155807.6094 | 18691.41797 | 114747.1797 | 0 | 60210.59375 | 152550.9531 | 51018.90234 | 123635.7969 | 14781.20801 | 20127.46875 |
| P08571 | 44281.23047 | 24299.92578 | 47487.57031 | 34793.12891 | 26150.58594 | 35119.19531 | 51497.69531 | 35848.58984 | 38011.08203 | 11786.36523 |
| P08603 | 174402.875 | 136935.0469 | 118284.1719 | 74779.69531 | 120419.6719 | 118032.9766 | 347212.0313 | 266349.5938 | 160958.6875 | 70233.8125 |
| P08697 | 52459.10156 | 33414.8125 | 50812.20703 | 55842.80469 | 74247.51563 | 53965.02734 | 303482.8125 | 74698.75781 | 54241.61328 | 51741.94531 |
| P08758 | 0 | 2310.864014 | 429.3574524 | 11625.91113 | 0 | 0 | 6209.536621 | 0 | 0 | 2442.72876 |
| P08779 | 14020.59863 | 50659.37891 | 9263.251953 | 13857.30566 | 27649.24219 | 6319.44043 | 43757.96875 | 60360.38281 | 7731.668457 | 25375.40234 |
| P09104 | 5974.812012 | 3236.273682 | 2229.984131 | 4289.29834 | 8220.147461 | 25490.62109 | 0 | 4155.237305 | 4183.026367 | 10498.33594 |
| P09211 | 5903.952637 | 6883.922852 | 3310.754883 | 12453.00586 | 3804.50708 | 2507.976563 | 0 | 0 | 0 | 7735.056641 |
| P09486 | 19381.53516 | 12051.17285 | 11172.2832 | 19528.58203 | 12657.30273 | 9443.651367 | 0 | 15570.65918 | 10697.0625 | 14460.85352 |
| P0C0L4 | 230434.1094 | 0 | 0 | 102113.6953 | 21199.33594 | 461049.3438 | 260092.8906 | 207926.7031 | 267593.9688 | 14870.64453 |
| P0C0L5 | 513253.5625 | 787888.5 | 609153 | 487139.3125 | 530588.0625 | 1477195.625 | 1117110.875 | 755871.1875 | 679334.625 | 670231.6875 |
| P0DJI8 | 0 | 801.8399048 | 3031.921631 | 1090.315918 | 5280.678711 | 6815.460938 | 0 | 10528.54297 | 20609.07422 | 0 |
| P0DOY2 | 1023181.688 | 1314452.875 | 1699240 | 1535624.75 | 1656124.375 | 811364.8125 | 3435002.75 | 1922781.25 | 1462218.5 | 2604506 |
| P10153 | 8169.912598 | 4214.999023 | 1237.109253 | 5478.282715 | 5001.731934 | 0 | 0 | 4586.883301 | 0 | 8057.581543 |
| P10253 | 4327.5 | 3766.4021 | 4598.185547 | 5262.550781 | 0 | 0 | 8022.631348 | 0 | 0 | 0 |
| P10451 | 581235 | 226566.9688 | 290757.3438 | 158178.3281 | 238644.9375 | 124110.5781 | 152416.4063 | 92600.13281 | 171080.0313 | 248625.2031 |
| P10523 | 6915.011719 | 2683.15332 | 6155.820801 | 3111.672852 | 12038.86426 | 69943.875 | 0 | 0 | 5006.669434 | 6491.165039 |
| P10586 | 3257.674072 | 3578.677246 | 6816.614258 | 4680.955078 | 2079.67749 | 0 | 0 | 2986.449707 | 36018.08594 | 0 |
| P10643 | 40220.72656 | 38080.34375 | 22463.42383 | 23944.67383 | 31068.47852 | 48330.90234 | 111718.2188 | 18909.48633 | 32872.86328 | 64650.94141 |
| P10645 | 8887.989258 | 10512.85742 | 19303.97852 | 7309.068359 | 6057.709473 | 0 | 0 | 7790.654297 | 4544.343262 | 0 |
| P10745 | 380069.3438 | 356243.7813 | 440701.0313 | 316949.125 | 252638.2969 | 259263.2031 | 192301.2969 | 92268.71875 | 105426.0703 | 24024.68555 |
| P10909 | 1909734.125 | 1344523.75 | 3011587 | 2186491.25 | 2530940 | 1348089.875 | 1658758 | 3018849.25 | 2590267.5 | 456600.7188 |
| P11021 | 8472.936523 | 4701.875 | 6919.496094 | 6046.499023 | 3618.981934 | 4607.82373 | 0 | 5938.495117 | 4647.039063 | 7365.293945 |
| P11047 | 3738.795898 | 2657.526855 | 2389.355957 | 3489.765137 | 0 | 5127.27002 | 0 | 0 | 1458.354492 | 3903.708008 |
| P11277 | 5938.469238 | 4515.452637 | 7529.17041 | 146.2311249 | 2042.425903 | 0 | 113089.9219 | 2221.219238 | 10273.26855 | 4684.094238 |
| P11279 | 14856.3457 | 14759.72656 | 19378.94727 | 11585.12012 | 11954.69824 | 0 | 0 | 0 | 6010.84375 | 16454.39453 |
| P12110 | 8327.540039 | 4211.496094 | 4068.305908 | 4555.856445 | 2848.594727 | 2839.148193 | 0 | 0 | 1172.947144 | 5362.084473 |
| P12111 | 6939.054199 | 4996.032227 | 4330.49707 | 11036.27246 | 5817.497559 | 11255.44531 | 21121.86914 | 10092.14258 | 8682.317383 | 18911.19727 |
| P12271 | 3732.068604 | 0 | 950.0075684 | 0 | 785.6003418 | 3247.962891 | 0 | 0 | 0 | 0 |
| P12277 | 2327.57666 | 0 | 2020.387085 | 0 | 2190.94873 | 75269.17969 | 0 | 4353.443359 | 1183.594849 | 0 |
| P12955 | 4696.0625 | 2152.365967 | 2284.187744 | 2739.851807 | 0 | 3838.755127 | 0 | 8793.823242 | 3849.006592 | 4271.584473 |
| P13473 | 72568.1875 | 27637.41992 | 31594.33594 | 55272.90625 | 42411.10938 | 7787.659668 | 0 | 8446.172852 | 35203.12891 | 66300.625 |
| P13645 | 19573.82813 | 163368.2656 | 14049.63477 | 13550.93262 | 82470.88281 | 20940.73828 | 115875.8438 | 144611.2813 | 21453.62109 | 46287.9375 |
| P13647 | 24001.61523 | 66124.75781 | 48422.51172 | 44964.30078 | 59811.48047 | 21667.20117 | 83371.04688 | 169291.4844 | 23356.33008 | 45512.25391 |
| P13671 | 31722.23242 | 34866.65625 | 37682.20313 | 28000.17383 | 53871.86328 | 61570.94141 | 219121.0781 | 106488.2969 | 70387.03125 | 35803.72656 |
| P13716 | 0 | 131.9149475 | 873.6084595 | 2155.824707 | 0 | 0 | 0 | 0 | 0 | 0 |
| P13796 | 3672.766357 | 3168.565186 | 4759.347656 | 9926.201172 | 7498.569336 | 8273.923828 | 0 | 14723.69043 | 4896.931641 | 6312.861816 |
| P14174 | 34291.99219 | 22379.3457 | 22227.17773 | 54783.625 | 9841.551758 | 29357.90625 | 0 | 10801.61914 | 12116.13184 | 59422.49219 |
| P14543 | 4091.968262 | 3260.381104 | 4570.033203 | 4269.986328 | 3858.205811 | 9129.816406 | 0 | 4767.584473 | 2842.39917 | 0 |
| P14550 | 0 | 1816.927612 | 1046.25415 | 3522.133301 | 0 | 0 | 0 | 0 | 0 | 0 |
| P14618 | 13826.36523 | 21597.9707 | 12776.23438 | 23655.59375 | 12620.60156 | 6899.548828 | 148253.8281 | 6406.790039 | 12541.86914 | 9116.269531 |
| P14625 | 7832.377441 | 2921.905029 | 4492.912109 | 4615.905762 | 4851.109375 | 0 | 0 | 7892.090332 | 4270.250977 | 0 |
| P15090 | 239.0130463 | 2290.298584 | 0 | 0 | 1010.841614 | 0 | 0 | 0 | 2469.739746 | 0 |
| P15121 | 0 | 884.3625488 | 1034.509644 | 1016.886963 | 0 | 0 | 0 | 0 | 0 | 0 |
| P15169 | 5403.469727 | 7066.028809 | 2481.063477 | 2727.026611 | 5318.645996 | 4463.666504 | 15356.66504 | 21432.87109 | 9673.438477 | 5292.424316 |
| P15291 | 18423.48242 | 4379.827148 | 15644.47461 | 16940.14648 | 12433.15918 | 10233.95313 | 4593.592773 | 6125.008301 | 10017.79297 | 5674.475586 |
| P15924 | 0 | 3839.402588 | 3914.160156 | 0 | 2395.562744 | 0 | 0 | 11270.59863 | 2162.812256 | 3937.176514 |
| P16035 | 17762.14258 | 17153.56445 | 20679.23047 | 17152.46875 | 12589.69531 | 31903.88281 | 41342.69922 | 10306.21875 | 15885.0625 | 22743.50195 |
| P16870 | 35149.44922 | 35861.13281 | 35341.09375 | 11568.55957 | 28655.17773 | 37288.80859 | 8998.666992 | 26607.34375 | 15370.92969 | 7480.370117 |
| P17050 | 9605.573242 | 6017.220215 | 6367.059082 | 7991.435547 | 0 | 0 | 0 | 0 | 0 | 0 |
| P17900 | 16334.75781 | 7079.421875 | 8179.541992 | 11051.52051 | 8136.001465 | 12845.60449 | 0 | 14543.89453 | 7146.210938 | 4577.91748 |
| P17931 | 2783.053467 | 0 | 2922.663818 | 0 | 3113.424561 | 0 | 0 | 0 | 0 | 0 |
| P18065 | 35817.02344 | 21032.2207 | 24872.60742 | 16330.1123 | 22270.21289 | 46619.64844 | 129863.5 | 30625.81445 | 29991.45898 | 16817.55859 |
| P18428 | 651.5921021 | 2066.472656 | 2643.093262 | 0 | 2257.263672 | 0 | 0 | 0 | 5124.474121 | 0 |
| P18669 | 5473.140625 | 2986.220703 | 3836.507324 | 4529.038574 | 4774.536133 | 15638.17773 | 26821.26953 | 5455.321289 | 7479.179688 | 6552.525391 |
| P19021 | 5950.561523 | 4119.406738 | 6003.617188 | 4537.03418 | 3308.395996 | 7380.348145 | 0 | 3316.549561 | 5023.067383 | 4494.864746 |
| P19022 | 31018.46094 | 30410.9375 | 19684.39648 | 29732.96484 | 30739.84375 | 37446.68359 | 0 | 16635.125 | 11087.80859 | 14096.92871 |
| P19320 | 4825.895508 | 7271.158203 | 6679.059082 | 6661.680664 | 8233.166992 | 17486.60742 | 0 | 14279.20996 | 5365.01709 | 0 |
| P19652 | 600053.75 | 595563.8125 | 595981.5625 | 632815.5 | 679549.5 | 274347.3125 | 565535.5 | 755634.5 | 806452.3125 | 1228145 |
| P19823 | 55464.12109 | 162450.3438 | 101761.75 | 28447.97656 | 110644.7422 | 256162.3438 | 316310.75 | 499196.0625 | 105163.4766 | 140928.2344 |
| P19827 | 73117 | 119828.8281 | 75265.86719 | 20528.61523 | 106948.0781 | 170809.6719 | 436201.0313 | 366210.25 | 80036.88281 | 98773.125 |
| P20774 | 14468.27246 | 18070.03125 | 18017.49414 | 10954.9834 | 22955.34766 | 40492.41016 | 28274.88672 | 22744.88086 | 15290.45117 | 26264.64258 |
| P20827 | 28290.32617 | 23468.23242 | 28799.15039 | 25429.1875 | 15709.11035 | 33344.92188 | 0 | 22603.58008 | 25017.11133 | 33633.42188 |
| P20849 | 16744.88281 | 24656.54102 | 32020.29883 | 19470.63672 | 10414.24023 | 9261.867188 | 0 | 5416.385254 | 5829.538574 | 12311.93457 |
| P20851 | 10102.13477 | 10033.82129 | 5539.324707 | 3440.47998 | 10911.12793 | 0 | 0 | 5244.933105 | 4164.528809 | 0 |
| P20908 | 2625.387939 | 0 | 1046.000366 | 844.2972412 | 0 | 2099.381592 | 0 | 0 | 0 | 0 |
| P21810 | 3732.811035 | 1943.141479 | 1478.427246 | 2441.240723 | 7723.132324 | 3700.237061 | 0 | 0 | 2112.063232 | 0 |
| P22304 | 10426.17383 | 10428 | 16791.35547 | 26207.19336 | 15055.2998 | 17311.93359 | 0 | 12807.18164 | 12646.67969 | 0 |
| P22314 | 1727.37207 | 0 | 291.3934326 | 4274.095215 | 0 | 7168.581055 | 4007.971924 | 2800.364014 | 6159.700684 | 6148.533203 |
| P22692 | 24708.70703 | 24154.95703 | 13277.2998 | 15297.28906 | 19705.64844 | 28094.77734 | 8511.427734 | 22939.625 | 25236.8125 | 35875.23047 |
| P22792 | 9747.748047 | 22146.95703 | 7183.607422 | 7141.093262 | 13170.63477 | 4848.62793 | 28930.125 | 27608.95703 | 25339.67188 | 7749.813965 |
| P22891 | 3552.862793 | 5661.969238 | 2654.821289 | 3132.168457 | 4072.296875 | 7764.678711 | 0 | 18247.17578 | 3073.659912 | 5238.663086 |
| P22914 | 23856 | 20075.76953 | 93145.21875 | 13422.38086 | 4131.057129 | 41266.76563 | 19585.99023 | 67494.10938 | 8210.412109 | 96277.40625 |
| P23083 | 11755.8291 | 8139.977539 | 6096.076172 | 0 | 0 | 7002.073242 | 0 | 6023.640625 | 8794.051758 | 0 |
| P23142 | 117819.5703 | 134800.1875 | 135854.125 | 100016.3047 | 86244.92969 | 138506.6406 | 161693.1406 | 115330.9531 | 76458.40625 | 174633.6406 |
| P23470 | 2713.747314 | 3153.688477 | 2393.903809 | 1963.654907 | 2776.480469 | 0 | 0 | 0 | 1750.230957 | 0 |
| P23471 | 8942.052734 | 11174.80176 | 15580.55078 | 8301.457031 | 7566.34082 | 0 | 11374.91895 | 6338.863281 | 5800.150391 | 2371.081055 |
| P24593 | 53409.10156 | 12528.07324 | 22311.78711 | 18876.73047 | 14179.02344 | 36240.19922 | 65239 | 40471.35938 | 20767.35742 | 15761.0166 |
| P25311 | 403970.0313 | 402741.0313 | 323641.625 | 591259.375 | 399221.4063 | 979881.125 | 1182668.5 | 1667048.5 | 864820 | 923039.6875 |
| P25774 | 1250.408569 | 2324.376221 | 285816.75 | 1495.128296 | 4280.850098 | 0 | 13471.1416 | 0 | 0 | 3237.419434 |
| P26038 | 3517.811523 | 3870.078125 | 3783.888916 | 5650.415039 | 9735.416992 | 8938.020508 | 0 | 10228.4502 | 6323.660645 | 5890.719727 |
| P26447 | 7389.916992 | 7969.850586 | 3517.64917 | 9064.373047 | 6434.590332 | 0 | 140955.5625 | 0 | 0 | 0 |
| P26572 | 580.9542236 | 1118.310791 | 1854.717651 | 0 | 0 | 0 | 0 | 0 | 0 | 0 |
| P26992 | 1836.095093 | 2111.160645 | 3995.107422 | 1356.473633 | 0 | 0 | 0 | 0 | 0 | 0 |
| P27169 | 12648.03027 | 26350.03516 | 15551.06543 | 9077.441406 | 31880.64063 | 22946.53516 | 147141.0313 | 11053.62891 | 22345.79688 | 27261.98633 |
| P28072 | 1872.708374 | 989.6257324 | 2755.25 | 3415.106201 | 0 | 0 | 0 | 0 | 0 | 0 |
| P29279 | 19862.10156 | 5488.70752 | 4926.271973 | 6246.993164 | 7739.039063 | 6442.712402 | 0 | 0 | 3019.770996 | 12158.38672 |
| P29401 | 2199.862305 | 6581.398438 | 4126.068359 | 14303.00781 | 760.4627075 | 6530.205566 | 49923.67188 | 5245.244141 | 4397.421387 | 7326.785645 |
| P29622 | 70289.89063 | 64822.41016 | 61272.38281 | 78384.85938 | 79509.375 | 50805.45313 | 86444.49219 | 66728.34375 | 85105.77344 | 100572.8281 |
| P30041 | 4838.59668 | 2735.268555 | 4538.518066 | 3423.295898 | 2035.678223 | 3121.412109 | 207449.7031 | 1990.605835 | 2035.068237 | 0 |
| P30043 | 20047.57813 | 20217.21875 | 17738.20117 | 20281.87891 | 0 | 67420.13281 | 35201.51172 | 54854.93359 | 23960.10156 | 0 |
| P30086 | 18989.69727 | 20909.47656 | 13704.60254 | 22344.01953 | 13422.21191 | 37950.21484 | 34221.87109 | 0 | 24410.58594 | 45830.80469 |
| P30101 | 0 | 4269.337891 | 1464.332764 | 1270.981934 | 0 | 0 | 0 | 0 | 19368.62891 | 0 |
| P30530 | 9492.009766 | 7524.796875 | 8989.328125 | 11132.49902 | 5227.253906 | 6948.74707 | 0 | 11692.5459 | 7308.737305 | 1444.092285 |
| P30740 | 2180.118896 | 2267.336182 | 2483.834961 | 0 | 2268.033691 | 0 | 0 | 0 | 2814.882568 | 0 |
| P30838 | 7964.209473 | 58331.76172 | 18637.93945 | 94464.91406 | 11061.00586 | 19782.08789 | 76804.99219 | 38062.10547 | 5616.526855 | 45244.48828 |
| P31025 | 25754.29297 | 11149.14844 | 12100.96094 | 23293.08984 | 26245.98828 | 12139.11133 | 38555.05078 | 17347.50391 | 8404.210938 | 57093.73438 |
| P31947 | 3446.789551 | 1585.710938 | 876.2445679 | 4774.575195 | 500.9874573 | 0 | 0 | 0 | 0 | 0 |
| P32119 | 51673.90625 | 14621.58398 | 69981.49219 | 40295.38281 | 13065.1543 | 19133.33594 | 13120.75 | 19204.25195 | 47892.95703 | 13198.39551 |
| P33908 | 10310.24805 | 6972.083984 | 9629.194336 | 10072.71973 | 16623.33398 | 30124.7793 | 15616.72559 | 27593.36523 | 8419.789063 | 0 |
| P34096 | 0 | 2251.98877 | 20878.04492 | 4858.266113 | 8835.576172 | 64741.65625 | 0 | 0 | 25990.40625 | 6741.589844 |
| P35527 | 74867.14844 | 109257.2109 | 22053.96484 | 37448.36328 | 211249.2813 | 28475.70117 | 84683.84375 | 1835124.625 | 47987.74219 | 37507.57813 |
| P35555 | 60380.69531 | 29739.5 | 38389.38672 | 37982.61719 | 21537.92188 | 39039.10938 | 48850.90625 | 27683.35352 | 20994.57813 | 33403.17188 |
| P35858 | 18910.55273 | 14932.98438 | 12097.23145 | 16195.71875 | 20629.94531 | 24357.36133 | 28171.66992 | 29628.00391 | 14307.2207 | 21400.63867 |
| P35908 | 34182.32813 | 219808.5781 | 63305.24219 | 40012.00391 | 161883.0156 | 30926.65234 | 139785.0469 | 565232.6875 | 28293.54297 | 33878.16016 |
| P35968 | 2888.712891 | 2328.599121 | 1522.733643 | 0 | 1476.568726 | 0 | 12557.35254 | 2825.568115 | 0 | 4368.678711 |
| P36222 | 40925.94531 | 104025.3984 | 99891.55469 | 36300.92188 | 154200.2188 | 67558.29688 | 582378.5 | 114645.6406 | 252625.7813 | 168504.4063 |
| P36955 | 2190212.75 | 2070571.375 | 3345058.5 | 1649695.625 | 1818531.5 | 1989269.875 | 3954897 | 2041896.25 | 1507909.625 | 1718796.75 |
| P37837 | 8776.99707 | 4439.891113 | 5709.007324 | 9245.724609 | 7857.969238 | 37818.20313 | 0 | 0 | 8431.294922 | 13215.375 |
| P39060 | 13594.78711 | 10354.08594 | 16917.98047 | 6332.07959 | 9287.764648 | 14816.91309 | 34236.625 | 16063.00684 | 12399.43652 | 4656.367676 |
| P40121 | 4099.987305 | 2106.120117 | 2238.378906 | 1970.930054 | 2084.083252 | 0 | 0 | 0 | 0 | 0 |
| P40189 | 4996.743164 | 3838.567871 | 5251.665039 | 3067.208496 | 2023.334473 | 0 | 0 | 0 | 0 | 0 |
| P41222 | 4729158 | 6339960.5 | 5119324.5 | 3562253 | 3205853 | 2519138.5 | 3282882.75 | 1368858.125 | 3358359.5 | 7456678 |
| P42785 | 4362.589355 | 4462.18457 | 4429.303711 | 3487.814941 | 4342.897461 | 0 | 0 | 0 | 0 | 0 |
| P43121 | 13185.72754 | 8279.355469 | 4846.127441 | 10311.37891 | 7740.790039 | 0 | 0 | 5309.214355 | 13749.15234 | 5866.939453 |
| P43234 | 10711.54883 | 8176.302246 | 10555.02637 | 6853.06543 | 4090.397217 | 0 | 0 | 0 | 0 | 5775.318848 |
| P43251 | 41365.92188 | 56285.66797 | 65374.44922 | 55745.97266 | 47090.26563 | 24630.98242 | 30285.82031 | 39769.77344 | 43548.00391 | 46215.76563 |
| P43320 | 3923.825684 | 4918.445313 | 15055.38086 | 27802.13477 | 4092.576904 | 19552.00391 | 0 | 55910.92969 | 0 | 17256.94531 |
| P43652 | 94713.625 | 65402.24219 | 120889.7188 | 193439.4063 | 142397.5 | 71925.125 | 136676.4375 | 160172.9219 | 176500.1094 | 326139.75 |
| P47972 | 4521.087402 | 2052.110107 | 3506.359863 | 1620.094971 | 6177.917969 | 0 | 94190.67188 | 65621.01563 | 19290.73828 | 13325.96387 |
| P48723 | 8270.172852 | 6898.085449 | 9363.75 | 6057.853027 | 4650.420898 | 7957.773438 | 0 | 9166.446289 | 4434.962891 | 0 |
| P49257 | 2615.971436 | 650.7451172 | 2209.404541 | 0 | 815.3040161 | 2348.684326 | 0 | 2045.587158 | 1286.932861 | 0 |
| P49788 | 1793.287842 | 1983.89563 | 2000.026123 | 4410.550781 | 3838.387451 | 1644.679932 | 0 | 0 | 1933.668213 | 0 |
| P49908 | 13084.7666 | 12636.61035 | 15466.22559 | 12233.00586 | 11639.76563 | 15945.75098 | 26255.90234 | 5908.285156 | 6128.991699 | 17561.37305 |
| P50395 | 2414.972656 | 2438.19043 | 3215.042236 | 2568.475586 | 983.2972412 | 7398.951172 | 0 | 0 | 1268.077881 | 0 |
| P51884 | 222020.5938 | 194239.3281 | 133682 | 307955.75 | 121014.7344 | 355684.6563 | 120165.3828 | 382436.2188 | 170701.1875 | 402171.8438 |
| P51888 | 25079.01367 | 25671.21289 | 38089.23828 | 15448.19824 | 17930.9043 | 26202.35547 | 196427.125 | 52248.58203 | 29975.66016 | 4263.851074 |
| P54802 | 2490.918945 | 2461.789063 | 1481.36438 | 639.6846924 | 1794.051514 | 0 | 0 | 0 | 0 | 1534.005737 |
| P55001 | 3537.92334 | 1984.96106 | 4588.117676 | 2646.251953 | 0 | 0 | 0 | 0 | 0 | 0 |
| P55058 | 7038.935059 | 9368.291992 | 13778.82617 | 11272.4502 | 15129.88867 | 17445.04102 | 16228.33984 | 17698.41211 | 12190.29004 | 0 |
| P55268 | 2742.834473 | 977.6629028 | 2513.080566 | 1750.660278 | 398.1860046 | 4530.074219 | 0 | 0 | 0 | 0 |
| P55283 | 2041.069702 | 2004.704224 | 3266.121338 | 0 | 0 | 0 | 0 | 0 | 0 | 0 |
| P55290 | 7508.268066 | 5551.683105 | 4013.880859 | 7370.080078 | 5004.505859 | 7009.564941 | 0 | 9701.451172 | 7687.069336 | 11606.77441 |
| P59665 | 15124.24902 | 10878.81445 | 12119.60254 | 9056.370117 | 15812.26172 | 38157.39453 | 98015.625 | 19504.67773 | 31094.49023 | 14404.58008 |
| P60174 | 18174.02734 | 21938.80078 | 17989.00195 | 39447.05859 | 13640.38281 | 55765.76953 | 25439.44336 | 21424.62109 | 22198.93359 | 33007.95313 |
| P60709 | 51590.85156 | 60847.60156 | 50673.59766 | 75298.71875 | 67571.10156 | 113684.6563 | 103774.1563 | 46993.72656 | 51471.96875 | 61863.49609 |
| P61626 | 14763.3125 | 27128.79297 | 15468.63477 | 10592.32129 | 11548.47852 | 41033.34375 | 142700.3438 | 53348.33203 | 34378.20313 | 13291.6875 |
| P61769 | 1314719.5 | 376610.6875 | 207498.2031 | 868577.6875 | 268619.0625 | 4235072 | 5839558.5 | 1170707.375 | 2243186 | 762695.5625 |
| P61812 | 6801.973145 | 7966.526855 | 10650.45508 | 5806.920898 | 3178.455322 | 6732.328613 | 30586.95898 | 8823.305664 | 4198.424805 | 0 |
| P61981 | 1769.487915 | 969.605835 | 1016.702698 | 2491.306641 | 3282.868652 | 0 | 0 | 0 | 0 | 2522.455811 |
| P62258 | 2185.630859 | 1150.047974 | 956.6383057 | 991.5626831 | 0 | 0 | 0 | 0 | 1076.28186 | 0 |
| P62805 | 1721.486084 | 2112.58374 | 4760.798828 | 2065.007324 | 0 | 2139.309082 | 25176.75586 | 0 | 86280.74219 | 4275.349121 |
| P62937 | 5171.20752 | 7924.963867 | 5882.745117 | 16551.28711 | 4815.101563 | 6463.709961 | 0 | 7928.86084 | 3996.372559 | 11276.68652 |
| P62942 | 2282.720947 | 1296.246704 | 1218.217773 | 0 | 0 | 0 | 0 | 0 | 0 | 0 |
| P62993 | 1329.276367 | 759.0279541 | 1303.269287 | 0 | 0 | 3216.886475 | 0 | 0 | 0 | 0 |
| P68871 | 1605118.25 | 1244314.875 | 1717675.5 | 2766949.75 | 635158.0625 | 387178.0938 | 137336.6406 | 237482.4844 | 1683623.25 | 475416.9375 |
| P69905 | 751539.1875 | 561736.3125 | 813535.6875 | 990170 | 389173 | 212612.6094 | 178235.9219 | 140989.8125 | 1450139.5 | 185319 |
| P78324 | 7685.844727 | 0 | 2835.794922 | 6738.194336 | 0 | 11734.63281 | 11221.95215 | 8011.863281 | 3062.467285 | 0 |
| P80108 | 11533.47949 | 13785.71582 | 3032.123535 | 3748.931885 | 7757.378418 | 5806.890625 | 10038.70801 | 10703.56055 | 16526.51172 | 1774.584229 |
| P80188 | 0 | 2521.916748 | 2404.98291 | 2578.052734 | 3310.636719 | 4753.618164 | 14262.21484 | 4799.739258 | 5432.56543 | 4968.836914 |
| P98160 | 24464.36719 | 29016.93945 | 31205.87109 | 32070.60156 | 30201.875 | 59870.28516 | 58399.53516 | 34838.78125 | 36306.39063 | 37744.31641 |
| P98164 | 20465.57227 | 11176.83301 | 61697.82422 | 10418.8252 | 54938.66016 | 13509.82324 | 29185.71484 | 9149.605469 | 9134.673828 | 8564.839844 |
| Q01459 | 0 | 27699.87109 | 35446.36328 | 29270.24219 | 13801.02539 | 36440.12109 | 0 | 0 | 9638.342773 | 17302.40039 |
| Q02413 | 0 | 7860.519043 | 2792.983398 | 1614.142334 | 0 | 640.8585205 | 0 | 7764.386719 | 0 | 5235.212402 |
| Q02487 | 17606.46875 | 8504.041016 | 11770.94629 | 12224.75195 | 7626.738281 | 16401.16602 | 0 | 10913.60547 | 14973.47266 | 37711.25781 |
| Q02809 | 1339.644043 | 1446.904663 | 1727.626465 | 3267.444336 | 0 | 1360.868652 | 0 | 0 | 0 | 0 |
| Q02818 | 16182.61035 | 10946.32813 | 17565.61523 | 5418.450684 | 10220.12695 | 10127.63965 | 0 | 9158.708008 | 7188.25293 | 6680.042969 |
| Q02985 | 15818.10254 | 3665.088379 | 954.2877808 | 14505.60156 | 3334.57373 | 24134.80273 | 26749.52344 | 115261.6719 | 31656.59766 | 0 |
| Q03591 | 33830.10156 | 42134.85938 | 37214.47266 | 27887.55469 | 19047.10938 | 17490.06836 | 39198.05859 | 37500.35547 | 34580.75391 | 30274.00586 |
| Q06033 | 2652.174316 | 10760.90918 | 6482.241211 | 0 | 8990.161133 | 9504.197266 | 19848.7207 | 23193.33398 | 5934.029785 | 4370.008301 |
| Q06481 | 15233.81641 | 20918.00195 | 23845.11914 | 10945.41406 | 10828.82324 | 9282.068359 | 0 | 0 | 2314.010742 | 0 |
| Q06830 | 83968.77344 | 26508.44531 | 97540.6875 | 74987.25 | 19674.30469 | 40230.22266 | 47723.49609 | 67205.125 | 73038.80469 | 28993.91602 |
| Q07507 | 15540.7666 | 16409.29102 | 10778.6875 | 8126.786133 | 136608.3125 | 35999.9375 | 44626.49609 | 5465.137207 | 14098.99023 | 443324.8438 |
| Q07954 | 0 | 1927.856201 | 1415.355957 | 0 | 1365.378662 | 0 | 0 | 0 | 0 | 0 |
| Q08380 | 46684.41016 | 15624.30469 | 26601.40039 | 31600.57813 | 21378.39844 | 44422.96875 | 47684.30078 | 24942.1875 | 28591.10156 | 46647.82031 |
| Q08397 | 7522.584961 | 2104.430664 | 5580.05127 | 3162.223877 | 2772.68335 | 8051.943848 | 11764.79199 | 4439.76416 | 4618.460938 | 0 |
| Q08629 | 10251.92383 | 8757.128906 | 14093.04883 | 8563.730469 | 5081.242676 | 15797.39063 | 28082.2832 | 13931.44531 | 4422.640137 | 0 |
| Q0P6D2 | 4709.727539 | 4633.585938 | 6164.199219 | 4081.748047 | 2000.373291 | 4428.996094 | 11289.4248 | 0 | 8361.272461 | 0 |
| Q10471 | 6738.320313 | 3044.955811 | 4523.594727 | 4483.812988 | 0 | 9278.455078 | 0 | 7112.424316 | 5868.492188 | 0 |
| Q10472 | 1493.375977 | 4352.431152 | 6267.612305 | 3903.45459 | 2278.44458 | 0 | 0 | 0 | 0 | 0 |
| Q12805 | 482419.2188 | 304212.2188 | 132577.625 | 118672.9453 | 379249.5313 | 651448.625 | 896978.1875 | 599196.4375 | 179592.3125 | 256152.4844 |
| Q12841 | 26370.99414 | 15544.30176 | 22851.22852 | 16623.79883 | 56840 | 4236.664063 | 43078.64844 | 4970.853516 | 12596.73535 | 13984.76367 |
| Q12860 | 5666.65625 | 7031.884766 | 10042.2168 | 3972.152344 | 6061.981934 | 12088.67578 | 0 | 6868.818848 | 7970.391113 | 4576.65332 |
| Q13228 | 4818.793457 | 5748.334961 | 7247.480469 | 9255.970703 | 3731.936768 | 4454.164063 | 0 | 1578.329224 | 6881.857422 | 11446.73145 |
| Q13231 | 0 | 24865.99219 | 10784.16699 | 0 | 7697.941406 | 12586.92773 | 13211.71191 | 0 | 452.0652161 | 1537.376343 |
| Q13332 | 3196.019531 | 2936.330811 | 4447.602051 | 2944.045898 | 0 | 0 | 0 | 0 | 0 | 0 |
| Q14055 | 15010.59766 | 13094.64648 | 19806.45703 | 12775.14453 | 11618.27539 | 14605.1875 | 12723.84766 | 10359.60059 | 12700.97656 | 9899.185547 |
| Q14112 | 611.7532959 | 1857.381592 | 2658.501953 | 6371.823242 | 0 | 0 | 0 | 0 | 2382.832275 | 0 |
| Q14118 | 40549.49219 | 28274.07031 | 39323.10156 | 37787.14063 | 23399.83594 | 39293.64844 | 21107.04102 | 56458.25 | 25433.87305 | 16313.34668 |
| Q14126 | 5790.365723 | 3789.819092 | 3846.210938 | 4374.541504 | 3679.752441 | 0 | 0 | 0 | 4804.779785 | 5240.11377 |
| Q14393 | 18193.63281 | 7375.586914 | 19935.39063 | 6544.737305 | 8336.714844 | 8344.188477 | 18765.21094 | 10405.24902 | 4063.033936 | 18796.74609 |
| Q14515 | 16970.62891 | 33271.73828 | 24187.77148 | 37630.23047 | 19473.67969 | 21843.75781 | 14813.70117 | 34833.80859 | 15048.6123 | 19809.92578 |
| Q14520 | 11325.47266 | 3249.462891 | 7602.88623 | 10808.21777 | 11695.26758 | 13854.13379 | 41631.58594 | 17031.98828 | 8846.330078 | 0 |
| Q14563 | 4942.820801 | 7395.250488 | 11888.31836 | 3974.936279 | 5819.283203 | 3040.056641 | 0 | 2351.309326 | 4008.967041 | 2416.635986 |
| Q14574 | 9129.935547 | 5746.167969 | 4039.772949 | 9587.709961 | 6272.87793 | 8929.886719 | 20965.13867 | 19723.22266 | 2790.489258 | 17777.86523 |
| Q14624 | 121023.5859 | 75931.20313 | 81772.34375 | 73746.26563 | 143645.1563 | 238590.9531 | 267734.625 | 394700.2813 | 147758.4375 | 59738.84766 |
| Q15113 | 40195.85156 | 38431.26563 | 22180.10352 | 27668.7207 | 22948.00781 | 52417.27734 | 32394.98633 | 36327.57031 | 22473.89844 | 70940.67969 |
| Q15223 | 4865.293457 | 7322.801758 | 10430.18652 | 8207.180664 | 5231.096191 | 2546.418701 | 0 | 3154.268555 | 2101.616943 | 0 |
| Q15293 | 0 | 2464.685059 | 1937.915039 | 3011.519775 | 0 | 0 | 0 | 0 | 2266.271484 | 0 |
| Q15582 | 37606.83594 | 21202.15039 | 27191.19141 | 41366.3125 | 32144.23828 | 54020.89063 | 78295.25781 | 15263.57129 | 20011.18359 | 82520.19531 |
| Q15782 | 0 | 4873.241211 | 4266.655273 | 1914.030273 | 4937.280762 | 0 | 0 | 0 | 1532.495972 | 0 |
| Q15828 | 5325.404785 | 4987.480957 | 5978.994141 | 3462.42041 | 5389.375977 | 16430.30273 | 34977.72656 | 30765.91016 | 25679.32617 | 9132.669922 |
| Q15904 | 13257.5127 | 12069.79785 | 14666.48242 | 6635.157227 | 10140.96484 | 6942.071289 | 0 | 6570.339844 | 6848.719238 | 0 |
| Q16270 | 360584.0625 | 310633.1563 | 349083.5313 | 408590.5 | 320300.625 | 505900.9063 | 743997.0625 | 366726.4063 | 307577.7813 | 657544.375 |
| Q16610 | 21849.89844 | 30180.65625 | 24318.2207 | 24194.04688 | 36192.625 | 13898.13867 | 53291.4375 | 23437.22461 | 17545.58398 | 47276.00781 |
| Q16661 | 3276.618408 | 1979.234009 | 0 | 0 | 0 | 10401.57031 | 8806.575195 | 6358.433105 | 4085.556885 | 0 |
| Q16706 | 1319.735352 | 1220.38208 | 1883.418823 | 1696.144409 | 1822.583008 | 4260.259766 | 0 | 0 | 0 | 0 |
| Q17R60 | 40125.48828 | 26235.35547 | 61947.02344 | 26376.58984 | 21475.29883 | 66000.74219 | 19815.0918 | 27923.4668 | 13390.94434 | 17378.75195 |
| Q495W5 | 3741.925781 | 3795.92041 | 0 | 7094.45752 | 2659.930176 | 5249.189453 | 0 | 0 | 2332.865234 | 644.6074829 |
| Q5JSG7 | 2045.817993 | 961.1974487 | 1026.091187 | 1585.143311 | 1 | 1.144147873 | 0 | 0 | 1 | 0 |
| Q5KU26 | 17587.61719 | 15387.18164 | 16722.66602 | 10470.875 | 12769.27148 | 3760.46875 | 0 | 700.4244385 | 8671.847656 | 23547.14844 |
| Q5T123 | 21721.75977 | 21151.41016 | 9454.464844 | 17732.66602 | 14227.50977 | 77188.50781 | 55094.60156 | 62645.12891 | 39729.36328 | 37466.01563 |
| Q63HQ2 | 1063.556641 | 644.1069336 | 1736.76001 | 0 | 0 | 0 | 0 | 0 | 0 | 0 |
| Q641Q3 | 6437.652832 | 2703.813965 | 4141.756836 | 0 | 2229.086182 | 5313.202637 | 0 | 0 | 0 | 0 |
| Q6E0U4 | 1017.758423 | 0 | 652.892395 | 0 | 0 | 1754.744995 | 0 | 2223.650391 | 2440.062256 | 0 |
| Q6EMK4 | 44985.20703 | 26627.78906 | 32296.95898 | 32242.65039 | 18842.76953 | 29110.66406 | 7694.064941 | 34681.28516 | 22794.95313 | 29928.47656 |
| Q6MZW2 | 2049.763672 | 3782.777588 | 6025.218262 | 5305.15918 | 3522.736328 | 1765.797241 | 0 | 0 | 0 | 0 |
| Q6PCB0 | 3619.456055 | 1858.972412 | 2256.749268 | 2969.177979 | 0 | 2541.903809 | 0 | 0 | 2045.857056 | 0 |
| Q6UWP8 | 1071.524902 | 589.1942139 | 1151.718994 | 0 | 0 | 0 | 0 | 3105.95166 | 1030.756836 | 0 |
| Q6UX71 | 26748.44531 | 25447.15625 | 28918.33594 | 30623.72461 | 21236.22461 | 21956.69141 | 0 | 25357.01367 | 19197.32617 | 18670.41992 |
| Q6ZMP0 | 9065.736328 | 8223.287109 | 18274.11914 | 3238.920654 | 1943.56311 | 7973.765137 | 0 | 13469.04004 | 2758.678955 | 0 |
| Q7Z3B1 | 12252.25977 | 7642.211914 | 9132.415039 | 10067.73047 | 6681.59375 | 8389.701172 | 0 | 11077.69922 | 7844.665527 | 4465.116211 |
| Q7Z7M0 | 0 | 1260.148193 | 1679.650879 | 923.829834 | 0 | 0 | 0 | 0 | 0 | 0 |
| Q86SR1 | 1971.636963 | 1247.707642 | 1279.443604 | 0 | 2054.579834 | 946.0557251 | 8388.509766 | 0 | 810.31073 | 694.8235474 |
| Q86UD1 | 20320.52148 | 4225.619141 | 15432.56543 | 2439.644287 | 27245.21484 | 11931.10449 | 0 | 18497.03516 | 12740.09961 | 0 |
| Q86X29 | 5898.814453 | 4109.166016 | 4415.710938 | 3140.480469 | 3399.371826 | 11112.8418 | 0 | 15385.11719 | 10256.16699 | 9056.822266 |
| Q86Y38 | 995.1813965 | 2193.209473 | 3469.563232 | 2161.963623 | 0 | 0 | 0 | 0 | 0 | 0 |
| Q86YZ3 | 2317.39624 | 3123.051758 | 3435.387695 | 1768.164673 | 8120.813477 | 0 | 0 | 33243.80469 | 966.9944458 | 0 |
| Q8IUX7 | 14829.62012 | 3110.533203 | 7823.438477 | 7734.015625 | 12804.33789 | 25389.00977 | 19733 | 8467.541992 | 5983.008789 | 0 |
| Q8IV08 | 9443.771484 | 6103.204102 | 7131.958496 | 8577.033203 | 3987.415527 | 3593.886719 | 0 | 0 | 2520.066406 | 0 |
| Q8IWU5 | 6425.178711 | 7797.888672 | 6960.94873 | 6229.087891 | 2894.648438 | 4927.728516 | 0 | 7325.925781 | 5827.772949 | 6636.084961 |
| Q8IWV2 | 2672.969238 | 2730.299805 | 4184.203125 | 2099.64502 | 2039.663086 | 927.4177856 | 0 | 2203.029053 | 0 | 0 |
| Q8IZJ3 | 68592.96875 | 54199.40625 | 55131.83984 | 54209.47266 | 54035.10938 | 78285.71875 | 111199.3906 | 42708.68359 | 52473.87891 | 67728.21094 |
| Q8N1N4 | 23851.88086 | 63515.13672 | 11877.42969 | 11305.33984 | 50863.5625 | 0 | 0 | 174361.3906 | 7186.415527 | 4866.858887 |
| Q8N3Z0 | 2774.177246 | 1657.826172 | 2484.380859 | 0 | 0 | 0 | 0 | 0 | 0 | 0 |
| Q8N436 | 2549.125488 | 1480.456299 | 3446.474854 | 1493.518555 | 0 | 977.3990479 | 0 | 0 | 0 | 0 |
| Q8N474 | 0 | 1373.513672 | 2502.508057 | 1651.473389 | 2760.745605 | 1417.225586 | 0 | 0 | 0 | 0 |
| Q8N475 | 33459.71875 | 8731.833984 | 21960.61914 | 9799.37207 | 20815.23828 | 15348.02734 | 17339.28516 | 12338.26758 | 15494.80273 | 3268.371094 |
| Q8NBJ4 | 12130.34375 | 10357.70508 | 12055.49512 | 8467.407227 | 9847.884766 | 14159.30078 | 0 | 17301.76172 | 9388.424805 | 15163.11621 |
| Q8NEV9 | 714.5741577 | 738.2523193 | 1445.683105 | 0 | 812.1056519 | 0 | 0 | 0 | 0 | 0 |
| Q8NFZ8 | 4401.78125 | 2414.203125 | 2834.310059 | 3748.970947 | 0 | 0 | 0 | 1284.605591 | 0 | 0 |
| Q8NHP8 | 6140.452148 | 11747.1582 | 9939.173828 | 6139.199219 | 2302.321777 | 19785.89453 | 0 | 27990.57617 | 9384.217773 | 19052.13672 |
| Q8TER0 | 1724.078857 | 1377.450073 | 2365.340332 | 1137.349121 | 1899.852539 | 3189.154053 | 0 | 0 | 0 | 1758.791992 |
| Q8WXD2 | 6819.848633 | 5741.357422 | 6290.91748 | 6094.144531 | 7194.407715 | 4233.106445 | 0 | 6140.109863 | 4816.983398 | 1811.940674 |
| Q8WY21 | 3597.86499 | 4417.155762 | 3378.124023 | 2906.127441 | 759.7661743 | 7922.557617 | 11461.27734 | 11319.62402 | 1932.571777 | 1335.233398 |
| Q92520 | 33284.96875 | 32674.64844 | 32591.03125 | 21166.1582 | 34355.125 | 72090.60156 | 127250.4141 | 30540.50195 | 32256.80859 | 34261.71875 |
| Q92673 | 1 | 432.0005493 | 1278.831787 | 1 | 0 | 0 | 0 | 0 | 1 | 2109.694092 |
| Q92743 | 11841.06445 | 6004.650879 | 8547.135742 | 10297.97754 | 6747.967773 | 5860.937012 | 9518.269531 | 12651.46191 | 4837.080566 | 4389.463379 |
| Q92765 | 102370.75 | 74003.89844 | 228150.7188 | 65705.41406 | 74018.88281 | 113445.2031 | 313920.875 | 160346.75 | 108016.4375 | 41061.97266 |
| Q92820 | 4020.799561 | 4854.536621 | 5062.099121 | 5188.879395 | 3199.905518 | 4365.797363 | 15310.90039 | 8736.384766 | 3579.730469 | 6599.34375 |
| Q92859 | 3906.700684 | 3000.263184 | 4365.651855 | 3271.975342 | 3280.151123 | 3347.731445 | 0 | 0 | 1128.317993 | 0 |
| Q92887 | 0 | 0 | 1724.489746 | 0 | 0 | 4862.344238 | 48080.89453 | 17745.35156 | 0 | 0 |
| Q96A11 | 4685.493164 | 0 | 6182.373047 | 2728.450195 | 0 | 0 | 0 | 0 | 0 | 0 |
| Q96FE5 | 0 | 3028.158936 | 4351.026367 | 3983.714844 | 0 | 0 | 0 | 0 | 0 | 0 |
| Q96FE7 | 4644.185547 | 2562.886719 | 3021.748291 | 5792.275391 | 1821.0177 | 5211.854004 | 0 | 0 | 3502.027344 | 0 |
| Q96GW7 | 1038.6521 | 2284.981201 | 2314.341064 | 2201.523682 | 0 | 0 | 0 | 0 | 0 | 0 |
| Q96HF1 | 27489.61914 | 6890.501465 | 20116.89453 | 5485.376953 | 10761.66016 | 11054.98438 | 36877.05078 | 13569.49414 | 11547.24805 | 0 |
| Q96JC1 | 0 | 4005.619141 | 13852.69727 | 27418.49219 | 0 | 11041.09961 | 0 | 0 | 19877.7832 | 24317.5293 |
| Q96JF0 | 1449.780273 | 3998.890869 | 4222.442871 | 0 | 0 | 5068.251465 | 0 | 0 | 0 | 0 |
| Q96JP9 | 33714.04688 | 11315.22266 | 33782.86719 | 21673.5 | 13010.3291 | 13945.00293 | 0 | 4568.711914 | 13930.28711 | 0 |
| Q96KG7 | 3069.775146 | 4310.110352 | 4477.017578 | 2934.966064 | 0 | 0 | 0 | 0 | 10979.23242 | 0 |
| Q96KN2 | 3720.915527 | 5108.405273 | 5855.188477 | 3028.410645 | 3573.976563 | 15961.86621 | 6866.598633 | 16007.95996 | 7697.56543 | 3379.595215 |
| Q96PD5 | 37070.68359 | 21451.16797 | 39231.36328 | 23342.73047 | 64431.40625 | 77219.02344 | 281166.875 | 52516.08594 | 54588.59375 | 31973.07617 |
| Q96PQ0 | 7015.465332 | 5917.575195 | 3378.864746 | 6756.590332 | 3609.32251 | 11695.69629 | 0 | 14298.4834 | 5826.455566 | 7984.79248 |
| Q96RW7 | 2114.028076 | 1627.160156 | 5301.354492 | 973.7490234 | 2412.073486 | 3966.595459 | 0 | 10693.97559 | 2321.923096 | 3851.837891 |
| Q96S96 | 11349.02734 | 17581.62891 | 19279.32031 | 24033.6875 | 3074.040283 | 31013.81641 | 18954.93945 | 34989.36719 | 24179.71484 | 19758.31641 |
| Q99574 | 16893.0918 | 24262.85352 | 18099.75781 | 18597.81055 | 15476.57227 | 26545.94336 | 15549.95703 | 21982.33203 | 14144.71973 | 15361.34961 |
| Q99784 | 16663.75391 | 11246.34766 | 14751.26563 | 9777.491211 | 10836.01367 | 4120.844238 | 0 | 9489.012695 | 10819.3291 | 2673.398438 |
| Q99972 | 37033.66797 | 28206.94141 | 33900.03516 | 42045.69141 | 33598.33984 | 68288.875 | 165668.8906 | 43095.88672 | 25498.19922 | 22157.28516 |
| Q99983 | 13224.4375 | 5659.779297 | 7335.482422 | 7787.509277 | 5977.601074 | 7353.59375 | 0 | 33141.28906 | 5365.401367 | 0 |
| Q9BQT9 | 4366.911621 | 2529.932861 | 3596.94873 | 4239.691406 | 1719.426392 | 1601.921509 | 0 | 0 | 6109.552734 | 0 |
| Q9BRK5 | 10955.58789 | 7048.750977 | 12880.59277 | 4382.900391 | 4989.439453 | 1606.621094 | 0 | 840.5473633 | 4770.744629 | 2751.817871 |
| Q9BS40 | 565.5058594 | 1969.650024 | 1582.807007 | 0 | 0 | 0 | 0 | 0 | 0 | 0 |
| Q9BTY2 | 12513.11133 | 9064.961914 | 10297.54004 | 9043.535156 | 8063.69043 | 3763.310303 | 19683.52344 | 6300.998047 | 3348.303711 | 6535.280273 |
| Q9BU40 | 5654.930664 | 3418.765381 | 5998.414063 | 1906.763916 | 0 | 0 | 76168.58594 | 0 | 6120.405273 | 0 |
| Q9BXJ0 | 5984.996094 | 1532.780762 | 6860.922852 | 15821.0166 | 1760.892334 | 0 | 0 | 0 | 2999.919922 | 6878.378418 |
| Q9BXP8 | 33006.63281 | 15205.77734 | 50112.91797 | 24615.62891 | 17248.34375 | 18490.30469 | 46229.20313 | 29941.12695 | 20936.23438 | 11169.58594 |
| Q9BYJ0 | 7794.45752 | 7790.578613 | 8179.693848 | 6989.768066 | 13567.65234 | 10556.40332 | 79349.83594 | 5708.106934 | 9081.40625 | 2642.791016 |
| Q9BZV3 | 7344.745117 | 26758.30664 | 16217.52148 | 39789.75 | 3513.07666 | 26752.92969 | 0 | 4920.857422 | 4941.957031 | 65757.73438 |
| Q9GZP0 | 2051.169678 | 2932.464844 | 3573.523438 | 3582.594727 | 1918.708496 | 3772.707275 | 0 | 0 | 2596.414551 | 0 |
| Q9GZX9 | 7748.385742 | 16028.7334 | 17931.26953 | 13321.88086 | 14712.11621 | 14991.08691 | 0 | 21164.91406 | 12746.21191 | 21710.95313 |
| Q9H2A7 | 22280.60547 | 14104.68262 | 22608.86133 | 21480.01758 | 7050.040039 | 21427.8418 | 0 | 20418.4707 | 12004.5791 | 10234.58984 |
| Q9H3G5 | 9730.098633 | 4960.462402 | 4182.776367 | 6227.75 | 1691.218994 | 3524.179688 | 0 | 0 | 1275.670898 | 9679.697266 |
| Q9H461 | 1662.093872 | 1706.716309 | 1743.267822 | 1182.356934 | 0 | 0 | 0 | 0 | 0 | 0 |
| Q9H4D0 | 12535.32617 | 7611.421387 | 11083.50195 | 7169.439941 | 9037.469727 | 11104.375 | 0 | 0 | 5718.144531 | 13448.88477 |
| Q9HAT2 | 10389.81152 | 6697.453125 | 10406.09863 | 4534.950195 | 5466.129883 | 5800.238281 | 0 | 4429.699219 | 5183.875977 | 4026.499023 |
| Q9HCB6 | 120645.4609 | 57311.71484 | 124696.3281 | 104900.9375 | 41700.13281 | 72100.46875 | 68328.67969 | 115126.5938 | 73865.28125 | 29731.79102 |
| Q9HCQ7 | 3168.111328 | 5567.498535 | 30428.24219 | 8245.192383 | 5277.823242 | 13290.14453 | 24685.96289 | 38752.75781 | 2674.876953 | 7708.731445 |
| Q9NPH3 | 9760.285156 | 6834.000977 | 6162.40918 | 10245.00488 | 10589.10156 | 16912.53516 | 0 | 12927.71484 | 4849.830566 | 8013.770508 |
| Q9NQ38 | 0 | 1647.170166 | 0 | 0 | 0 | 4661.840332 | 0 | 7691.413086 | 2814.244629 | 0 |
| Q9NR34 | 2343.240967 | 1439.488159 | 3681.752441 | 2597.893066 | 0 | 3276.456543 | 0 | 0 | 0 | 0 |
| Q9NRN5 | 11360.5 | 8607.646484 | 11480.63086 | 15825.02832 | 8670.517578 | 20637.06055 | 0 | 9529.97168 | 10069.9541 | 15490.5293 |
| Q9NRR1 | 9595.349609 | 8580.212891 | 4759.474121 | 18630.34961 | 9186.566406 | 13662.54297 | 90034.4375 | 5460.79834 | 7231.423828 | 53913.83203 |
| Q9NZ08 | 2310.790527 | 2463.896729 | 3752.466064 | 2504.954102 | 0 | 0 | 0 | 0 | 2710.814697 | 0 |
| Q9NZP8 | 6009.331055 | 2787.747314 | 2599.525146 | 4441.608887 | 5200.857422 | 3187.902344 | 8190.178223 | 7478.435547 | 7311.166992 | 7597.291992 |
| Q9UBM4 | 362211.125 | 221476.9844 | 295093.6875 | 181304.5938 | 208443.9219 | 301960.3438 | 645784.3125 | 197771.8281 | 214927.6719 | 52202.14063 |
| Q9UBR2 | 31927.61523 | 26358.59766 | 31307.25781 | 21867.55469 | 21788.5625 | 46635.60156 | 69193.26563 | 18944.38281 | 23847.92188 | 25134.50195 |
| Q9UBX1 | 3255.508057 | 1606.163208 | 1936.156616 | 1051.040894 | 1423.53479 | 0 | 0 | 5100.852539 | 1110.515747 | 0 |
| Q9UFW8 | 153404.0313 | 245656.4063 | 364686.25 | 58393.875 | 26172.79102 | 114253.2109 | 893125.25 | 633016.3125 | 539592.0625 | 45528.15234 |
| Q9UGM5 | 22714.17383 | 17724.0957 | 29103.39453 | 33173.44922 | 28781.53711 | 30609.73047 | 30022.50391 | 33823.38281 | 45907.26953 | 41762.6875 |
| Q9UHG2 | 31873.22461 | 25949.66406 | 36466.00391 | 36861.55859 | 32801.42188 | 88439.67188 | 27747.55273 | 38746.45313 | 29789.20898 | 38339.67969 |
| Q9UHL4 | 21091.36914 | 14361.78613 | 14112.08887 | 13202.43945 | 0 | 12753.81445 | 0 | 6504.359375 | 0 | 10681.31641 |
| Q9UJJ9 | 3370.065674 | 1604.9646 | 4338.352539 | 5285.216309 | 2428.188965 | 9351.951172 | 0 | 10061.21973 | 2651.327148 | 0 |
| Q9UM22 | 5036.73291 | 8748.337891 | 9384.188477 | 723.0960083 | 0 | 2921.560547 | 20101.05273 | 0 | 0 | 0 |
| Q9UMX5 | 878.0031738 | 419.9239502 | 1198.65979 | 0 | 0 | 0 | 0 | 0 | 349.1754761 | 0 |
| Q9UNA0 | 8260.192383 | 12467.16602 | 6157.605957 | 54903.36328 | 1918.324463 | 1969.304321 | 0 | 759.6029053 | 33972.60938 | 57217.51953 |
| Q9UNW1 | 4128.338379 | 5056.391113 | 7357.862793 | 6415.618652 | 0 | 0 | 0 | 0 | 0 | 0 |
| Q9Y240 | 3149.167969 | 1903.845825 | 2476.56958 | 4059.196533 | 0 | 4349.072266 | 0 | 1429.273682 | 2359.824951 | 0 |
| Q9Y279 | 15087.3252 | 15422.1582 | 15991.75 | 15412.47168 | 8729.573242 | 38957.83594 | 0 | 45087.28125 | 26413.61523 | 13420.20703 |
| Q9Y5W5 | 33994.94141 | 20307.91406 | 37367.13281 | 18356.83789 | 14146.62012 | 21851.66797 | 32841.51953 | 18790.51953 | 15057.36328 | 4752.540527 |
| Q9Y5Y7 | 3794.615723 | 6918.47998 | 5285.813477 | 12336.62695 | 3991.736816 | 12466.82715 | 0 | 18357.01563 | 7633.584961 | 15571.76953 |
| Q9Y646 | 17331.30078 | 19713.49219 | 66286.84375 | 89082.17969 | 13198.01758 | 101406.9609 | 9810.985352 | 17348.25977 | 99053.03125 | 32108.99219 |
| Q9Y6C2 | 2170.983643 | 1174.853027 | 3616.47583 | 1454.037598 | 2482.67749 | 3258.869873 | 0 | 0 | 0 | 0 |
| Q9Y6R7 | 122414.9219 | 36716.51172 | 228635 | 71248.65625 | 45816.5 | 69543.48438 | 57504.28516 | 139841.4375 | 48647.0625 | 35450.97266 |
