# Supplementary material for: Pursuing Diabetic Nephropathy through Aqueous Humor Proteomics Analysis
Source: Oxid Med Cell Longev. 2022 Sep 29;2022:5945828. doi: 10.1155/2022/5945828 (PMC9537621; doi:10.1155/2022/5945828)
Supplement: Supplementary 2 — Table S2: Protein-protein interaction analysis results using the STRING database. [file 5945828.f2.doc]

**Table S2. Protein-protein interaction analysis results using the STRING database**

| #node1 | node2 | node1_string_id | node2_string_id | neighborhood_on_chromosome | gene_fusion | phylogenetic_cooccurrence | homology | coexpression | experimentally_determined_interaction | database_annotated | automated_textmining | combined_score |
| --- | --- | --- | --- | --- | --- | --- | --- | --- | --- | --- | --- | --- |
| APOH | C8A | 9606.ENSP00000205948 | 9606.ENSP00000354458 | 0 | 0 | 0 | 0 | 0.347 | 0 | 0 | 0.135 | 0.411 |
| APOH | CST6 | 9606.ENSP00000205948 | 9606.ENSP00000311313 | 0 | 0 | 0 | 0 | 0 | 0 | 0 | 0.438 | 0.438 |
| APOH | RBP4 | 9606.ENSP00000205948 | 9606.ENSP00000360522 | 0 | 0 | 0 | 0 | 0.283 | 0 | 0 | 0.358 | 0.52 |
| APOH | F10 | 9606.ENSP00000205948 | 9606.ENSP00000364709 | 0 | 0 | 0 | 0 | 0.183 | 0.213 | 0 | 0.314 | 0.52 |
| APOH | AZGP1 | 9606.ENSP00000205948 | 9606.ENSP00000292401 | 0 | 0 | 0 | 0 | 0.188 | 0 | 0 | 0.467 | 0.549 |
| APOH | C3 | 9606.ENSP00000205948 | 9606.ENSP00000245907 | 0 | 0 | 0 | 0 | 0.111 | 0 | 0 | 0.635 | 0.662 |
| APOH | PLG | 9606.ENSP00000205948 | 9606.ENSP00000308938 | 0 | 0 | 0 | 0 | 0.187 | 0.472 | 0 | 0.694 | 0.857 |
| APOH | APOM | 9606.ENSP00000205948 | 9606.ENSP00000365081 | 0 | 0 | 0 | 0 | 0.133 | 0 | 0.72 | 0.517 | 0.872 |
| APOH | SERPINC1 | 9606.ENSP00000205948 | 9606.ENSP00000356671 | 0 | 0 | 0 | 0 | 0.819 | 0.057 | 0 | 0.71 | 0.946 |
| APOM | C3 | 9606.ENSP00000365081 | 9606.ENSP00000245907 | 0 | 0 | 0 | 0 | 0.064 | 0 | 0 | 0.598 | 0.608 |
| APOM | PLG | 9606.ENSP00000365081 | 9606.ENSP00000308938 | 0 | 0 | 0 | 0 | 0.641 | 0 | 0 | 0.09 | 0.659 |
| APOM | SERPINC1 | 9606.ENSP00000365081 | 9606.ENSP00000356671 | 0 | 0 | 0 | 0 | 0.281 | 0 | 0 | 0.621 | 0.716 |
| AZGP1 | RBP4 | 9606.ENSP00000292401 | 9606.ENSP00000360522 | 0 | 0 | 0 | 0 | 0.16 | 0 | 0 | 0.543 | 0.599 |
| BTD | GNS | 9606.ENSP00000400995 | 9606.ENSP00000258145 | 0 | 0 | 0 | 0 | 0.062 | 0.392 | 0 | 0 | 0.405 |
| BTD | SIAE | 9606.ENSP00000400995 | 9606.ENSP00000263593 | 0 | 0 | 0 | 0 | 0.062 | 0.4 | 0 | 0 | 0.413 |
| BTD | TPP1 | 9606.ENSP00000400995 | 9606.ENSP00000299427 | 0 | 0 | 0 | 0 | 0 | 0 | 0 | 0.711 | 0.711 |
| C3 | LCN2 | 9606.ENSP00000245907 | 9606.ENSP00000362108 | 0 | 0 | 0 | 0 | 0.09 | 0 | 0 | 0.38 | 0.411 |
| C3 | F10 | 9606.ENSP00000245907 | 9606.ENSP00000364709 | 0 | 0 | 0 | 0 | 0.106 | 0.134 | 0 | 0.349 | 0.452 |
| C3 | RBP4 | 9606.ENSP00000245907 | 9606.ENSP00000360522 | 0 | 0 | 0 | 0 | 0.147 | 0 | 0 | 0.392 | 0.46 |
| C3 | SERPINC1 | 9606.ENSP00000245907 | 9606.ENSP00000356671 | 0 | 0 | 0 | 0 | 0.134 | 0 | 0 | 0.559 | 0.602 |
| C3 | PLG | 9606.ENSP00000245907 | 9606.ENSP00000308938 | 0 | 0 | 0 | 0 | 0.147 | 0.156 | 0.8 | 0.696 | 0.95 |
| C3 | C8A | 9606.ENSP00000245907 | 9606.ENSP00000354458 | 0 | 0 | 0 | 0 | 0.118 | 0.078 | 0 | 0.962 | 0.966 |
| C8A | SERPINC1 | 9606.ENSP00000354458 | 9606.ENSP00000356671 | 0 | 0 | 0 | 0 | 0.696 | 0 | 0 | 0.202 | 0.747 |
| CDH2 | CHL1 | 9606.ENSP00000269141 | 9606.ENSP00000256509 | 0 | 0 | 0 | 0 | 0.061 | 0 | 0 | 0.762 | 0.768 |
| CDH2 | NECTIN1 | 9606.ENSP00000269141 | 9606.ENSP00000264025 | 0 | 0 | 0 | 0 | 0.062 | 0.058 | 0 | 0.526 | 0.545 |
| CDH2 | VCAN | 9606.ENSP00000269141 | 9606.ENSP00000265077 | 0 | 0 | 0 | 0 | 0.091 | 0 | 0 | 0.38 | 0.412 |
| CDH2 | SEMA3A | 9606.ENSP00000269141 | 9606.ENSP00000265362 | 0 | 0 | 0 | 0 | 0.107 | 0.057 | 0 | 0.413 | 0.462 |
| CDH2 | NRCAM | 9606.ENSP00000269141 | 9606.ENSP00000368314 | 0 | 0 | 0 | 0 | 0.098 | 0 | 0 | 0.458 | 0.491 |
| CDH2 | NCAM1 | 9606.ENSP00000269141 | 9606.ENSP00000480132 | 0 | 0 | 0 | 0 | 0.105 | 0.07 | 0 | 0.884 | 0.896 |
| CHL1 | NCAM1 | 9606.ENSP00000256509 | 9606.ENSP00000480132 | 0 | 0 | 0 | 0.549 | 0.198 | 0.148 | 0 | 0.922 | 0.583 |
| CHL1 | SEMA3A | 9606.ENSP00000256509 | 9606.ENSP00000265362 | 0 | 0 | 0 | 0 | 0 | 0.109 | 0 | 0.562 | 0.594 |
| CHL1 | PTPRZ1 | 9606.ENSP00000256509 | 9606.ENSP00000377047 | 0 | 0 | 0 | 0 | 0.134 | 0.154 | 0 | 0.759 | 0.809 |
| COL6A1 | COL6A2 | 9606.ENSP00000355180 | 9606.ENSP00000300527 | 0 | 0 | 0 | 0.828 | 0.964 | 0 | 0.9 | 0.955 | 0.996 |
| COL6A1 | THBS4 | 9606.ENSP00000355180 | 9606.ENSP00000339730 | 0 | 0 | 0 | 0 | 0.064 | 0.056 | 0.5 | 0.442 | 0.72 |
| COL6A1 | COL9A1 | 9606.ENSP00000355180 | 9606.ENSP00000349790 | 0 | 0 | 0 | 0.617 | 0.062 | 0.285 | 0.3 | 0.454 | 0.573 |
| COL6A1 | NCAM1 | 9606.ENSP00000355180 | 9606.ENSP00000480132 | 0 | 0 | 0 | 0 | 0 | 0 | 0.6 | 0.18 | 0.658 |
| COL6A2 | COL9A1 | 9606.ENSP00000300527 | 9606.ENSP00000349790 | 0 | 0 | 0 | 0.631 | 0 | 0.288 | 0.3 | 0.137 | 0.499 |
| COL6A2 | THBS4 | 9606.ENSP00000300527 | 9606.ENSP00000339730 | 0 | 0 | 0 | 0 | 0.062 | 0.056 | 0.5 | 0.254 | 0.625 |
| COL6A2 | NCAM1 | 9606.ENSP00000300527 | 9606.ENSP00000480132 | 0 | 0 | 0 | 0 | 0 | 0 | 0.6 | 0.182 | 0.658 |
| COL9A1 | THBS4 | 9606.ENSP00000349790 | 9606.ENSP00000339730 | 0 | 0 | 0 | 0 | 0 | 0.056 | 0.5 | 0.147 | 0.562 |
| COL9A1 | NCAM1 | 9606.ENSP00000349790 | 9606.ENSP00000480132 | 0 | 0 | 0 | 0 | 0 | 0 | 0.6 | 0.117 | 0.631 |
| F10 | PLG | 9606.ENSP00000364709 | 9606.ENSP00000308938 | 0 | 0 | 0 | 0.658 | 0.162 | 0.27 | 0 | 0.787 | 0.531 |
| F10 | SERPINC1 | 9606.ENSP00000364709 | 9606.ENSP00000356671 | 0 | 0 | 0 | 0 | 0.186 | 0.816 | 0.9 | 0.987 | 0.999 |
| FUCA2 | PLG | 9606.ENSP00000002165 | 9606.ENSP00000308938 | 0 | 0 | 0 | 0 | 0 | 0 | 0 | 0.624 | 0.624 |
| GNS | TPP1 | 9606.ENSP00000258145 | 9606.ENSP00000299427 | 0 | 0 | 0 | 0 | 0.208 | 0 | 0 | 0.276 | 0.402 |
| GNS | PSAP | 9606.ENSP00000258145 | 9606.ENSP00000378394 | 0 | 0 | 0 | 0 | 0.188 | 0 | 0 | 0.342 | 0.443 |
| GNS | SIAE | 9606.ENSP00000258145 | 9606.ENSP00000263593 | 0 | 0 | 0 | 0 | 0 | 0.391 | 0 | 0.252 | 0.525 |
| LCN2 | RBP4 | 9606.ENSP00000362108 | 9606.ENSP00000360522 | 0 | 0 | 0 | 0 | 0.062 | 0 | 0 | 0.594 | 0.602 |
| NCAM1 | NECTIN1 | 9606.ENSP00000480132 | 9606.ENSP00000264025 | 0 | 0 | 0 | 0 | 0 | 0 | 0 | 0.687 | 0.687 |
| NCAM1 | SEMA3A | 9606.ENSP00000480132 | 9606.ENSP00000265362 | 0 | 0 | 0 | 0 | 0.062 | 0.103 | 0 | 0.367 | 0.421 |
| NCAM1 | NRCAM | 9606.ENSP00000480132 | 9606.ENSP00000368314 | 0 | 0 | 0 | 0.56 | 0.184 | 0.148 | 0 | 0.755 | 0.513 |
| NCAM1 | PTPRZ1 | 9606.ENSP00000480132 | 9606.ENSP00000377047 | 0 | 0 | 0 | 0 | 0.159 | 0.087 | 0 | 0.877 | 0.898 |
| NRCAM | SEMA3A | 9606.ENSP00000368314 | 9606.ENSP00000265362 | 0 | 0 | 0 | 0 | 0 | 0.109 | 0 | 0.699 | 0.72 |
| NRCAM | PTPRZ1 | 9606.ENSP00000368314 | 9606.ENSP00000377047 | 0 | 0 | 0 | 0 | 0.157 | 0.154 | 0 | 0.841 | 0.878 |
| NUCB1 | SDF4 | 9606.ENSP00000385923 | 9606.ENSP00000353094 | 0 | 0 | 0 | 0 | 0.174 | 0 | 0 | 0.574 | 0.633 |
| NUCB1 | PSAP | 9606.ENSP00000385923 | 9606.ENSP00000378394 | 0 | 0 | 0 | 0 | 0.67 | 0 | 0 | 0.118 | 0.696 |
| PLG | RBP4 | 9606.ENSP00000308938 | 9606.ENSP00000360522 | 0 | 0 | 0 | 0 | 0.187 | 0 | 0 | 0.325 | 0.427 |
| PLG | UBA1 | 9606.ENSP00000308938 | 9606.ENSP00000338413 | 0 | 0 | 0 | 0 | 0 | 0 | 0.9 | 0 | 0.9 |
| PLG | SERPINC1 | 9606.ENSP00000308938 | 9606.ENSP00000356671 | 0 | 0 | 0 | 0 | 0.674 | 0.391 | 0 | 0.917 | 0.982 |
| PLXDC2 | SEZ6 | 9606.ENSP00000366460 | 9606.ENSP00000312942 | 0 | 0 | 0 | 0 | 0 | 0 | 0 | 0.433 | 0.433 |
| PSAP | TPP1 | 9606.ENSP00000378394 | 9606.ENSP00000299427 | 0 | 0 | 0 | 0 | 0.125 | 0 | 0 | 0.407 | 0.459 |
| PTPRZ1 | VCAN | 9606.ENSP00000377047 | 9606.ENSP00000265077 | 0 | 0 | 0 | 0 | 0.069 | 0 | 0 | 0.771 | 0.778 |
| SEMA3A | VCAN | 9606.ENSP00000265362 | 9606.ENSP00000265077 | 0 | 0 | 0 | 0 | 0.065 | 0 | 0 | 0.47 | 0.483 |
