# Supplementary material for: Pursuing Diabetic Nephropathy through Aqueous Humor Proteomics Analysis
Source: Oxid Med Cell Longev. 2022 Sep 29;2022:5945828. doi: 10.1155/2022/5945828 (PMC9537621; doi:10.1155/2022/5945828)
Supplement: Supplementary 3 — Table S3: Statistically significant upstream regulators from IPA. [file 5945828.f3.doc]

**Table S3. Statistically significant upstream regulators from IPA**

| Upstream Regulator | Molecule Type | p-value of overlap | Target molecules in dataset |
| --- | --- | --- | --- |
| IL1B | cytokine | 0.0048 | C3,CDH2,PLXDC2,SEMA3A |
| TFEB | transcription regulator | 0.0000247 | GNS,PSAP,TPP1 |
| PPARA | ligand-dependent nuclear receptor | 0.000384 | APOM,C3,RBP4 |
| SPDEF | transcription regulator | 0.000404 | CDH2,COL6A1,COL6A2 |
| JINK1/2 | group | 0.000463 | CDH2,PLG |
| DNAJB6 | transcription regulator | 0.000622 | AZGP1,CDH2 |
| CHI3L1 | enzyme | 0.00135 | COL6A1,COL6A2 |
| Jnk | group | 0.00141 | CDH2,PLG,VCAN |
| Akt | group | 0.00165 | CDH2,LCN2,PLG |
| SOX4 | transcription regulator | 0.00189 | CDH2,SEMA3A |
| GABARAP | transporter | 0.00235 | PSAP,TPP1 |
| GABARAPL1 | other | 0.00235 | PSAP,TPP1 |
| GABARAPL2 | other | 0.00235 | PSAP,TPP1 |
| TGFB2 | growth factor | 0.00235 | COL6A2,VCAN |
| CASP2 | peptidase | 0.00248 | NCAM1 |
| CASP9 | peptidase | 0.00248 | NCAM1 |
| PDC | other | 0.00248 | RBP3 |
| ZNF239 | transcription regulator | 0.00248 | RBP3 |
| ETV5 | transcription regulator | 0.00381 | CDH2,NRCAM |
| BCAN | other | 0.00496 | CDH2 |
| PGRMC1 | transmembrane receptor | 0.00496 | LCN2 |
| RPS4Y1 | other | 0.00496 | CDH2 |
| miR-194-5p (miRNAs w/seed GUAACAG) | mature microrna | 0.00496 | CDH2 |
| CDH1 | other | 0.00512 | CDH2,NCAM1 |
| TWIST1 | transcription regulator | 0.00634 | C3,CDH2 |
| CDH3 | other | 0.00743 | CDH2 |
| DDR1 | kinase | 0.00743 | CDH2 |
| FER | kinase | 0.00743 | B4GAT1 |
| PTPN23 | phosphatase | 0.00743 | CDH2 |
| TRMT13 | other | 0.00743 | CDH2 |
| TXNRD1 | enzyme | 0.00743 | GPX3 |
| XRCC5 | enzyme | 0.00743 | SEMA3A |
| APOC1 | transporter | 0.0099 | CDH2 |
| BAALC | other | 0.0099 | CDH2 |
| CCN4 | other | 0.0099 | CDH2 |
| DEPTOR | other | 0.0099 | CDH2 |
| ETV4 | transcription regulator | 0.0099 | B4GALT1 |
| GGCT | enzyme | 0.0099 | CDH2 |
| HIF1A-AS2 | other | 0.0099 | CDH2 |
| HOXA-AS3 | other | 0.0099 | CDH2 |
| LINC00887 | other | 0.0099 | CDH2 |
| MFAP2 | other | 0.0099 | VCAN |
| NR2F1-AS1 | other | 0.0099 | CDH2 |
| OPRM1 | g-protein coupled receptor | 0.0099 | CDH2 |
| PCCA-DT | other | 0.0099 | CDH2 |
| RGCC | other | 0.0099 | CDH2 |
| CTNND1 | other | 0.0124 | CDH2 |
| DLGAP1 | other | 0.0124 | CDH2 |
| EHD1 | other | 0.0124 | CDH2 |
| GALNT4 | enzyme | 0.0124 | CDH2 |
| MAD2L2 | enzyme | 0.0124 | CDH2 |
| MSX2 | transcription regulator | 0.0124 | CDH2 |
| NOCT | transcription regulator | 0.0124 | PTPRZ1 |
| PPP3CA | phosphatase | 0.0124 | CDH2 |
| Tnf receptor | group | 0.0124 | C3 |
| WBP11 | phosphatase | 0.0124 | CDH2 |
| miR-380-3p (miRNAs w/seed AUGUAAU) | mature microrna | 0.0124 | CDH2 |
| mir-612 | microrna | 0.0124 | CDH2 |
| estrogen receptor | group | 0.014 | C3,COL6A1,COL6A2 |
| BMP7 | growth factor | 0.0142 | CDH2,VCAN |
| EZH2 | transcription regulator | 0.0144 | C3,CDH2,LCN2 |
| CBR3-AS1 | other | 0.0148 | CDH2 |
| CPXM1 | peptidase | 0.0148 | COL6A1 |
| ENO1 | enzyme | 0.0148 | CDH2 |
| EOGT | enzyme | 0.0148 | GPX3 |
| IRF6 | transcription regulator | 0.0148 | CDH2 |
| LINC00842 | other | 0.0148 | CDH2 |
| MAPK7 | kinase | 0.0148 | CDH2 |
| NET1 | other | 0.0148 | CDH2 |
| NR0B2 | ligand-dependent nuclear receptor | 0.0148 | APOM |
| NR1H2 | ligand-dependent nuclear receptor | 0.0148 | C3 |
| S100A10 | other | 0.0148 | PLG |
| SNHG29 | other | 0.0148 | CDH2 |
| CTNNB1 | transcription regulator | 0.0153 | CDH2,NRCAM,VCAN |
| ARHGAP4 | other | 0.0173 | CDH2 |
| BCAR4 | other | 0.0173 | CDH2 |
| BIN1 | other | 0.0173 | CDH2 |
| C3AR1 | g-protein coupled receptor | 0.0173 | C3 |
| CUL7 | enzyme | 0.0173 | CDH2 |
| GJA1 | transporter | 0.0173 | CDH2 |
| MIA | other | 0.0173 | CDH2 |
| NRDE2 | other | 0.0173 | TPP1 |
| SENP7 | peptidase | 0.0173 | CDH2 |
| ST6GALNAC1 | enzyme | 0.0173 | CDH2 |
| THBS4 | other | 0.0173 | CDH2 |
| mir-24 | microrna | 0.0173 | PSAP |
| mir-338 | microrna | 0.0173 | CDH2 |
| DDB2 | other | 0.0197 | SEMA3A |
| HMMR | transmembrane receptor | 0.0197 | CDH2 |
| KDM4B | enzyme | 0.0197 | LCN2 |
| KRT17 | other | 0.0197 | CDH2 |
| MGAT3 | enzyme | 0.0197 | CDH2 |
| PTBP3 | other | 0.0197 | CDH2 |
| SSTR2 | g-protein coupled receptor | 0.0197 | CDH2 |
| USP2 | peptidase | 0.0197 | SERPINC1 |
| HDAC1 | transcription regulator | 0.0206 | COL9A1,LCN2 |
| AR | ligand-dependent nuclear receptor | 0.0215 | AZGP1,CDH2,VCAN |
| PTPRR | phosphatase | 0.022 | COL6A1,NUCB1 |
| FZD8 | g-protein coupled receptor | 0.0221 | VCAN |
| GALNT3 | enzyme | 0.0221 | CDH2 |
| KDM6B | enzyme | 0.0221 | CDH2 |
| LRP1 | transmembrane receptor | 0.0221 | CDH2 |
| MZF1 | transcription regulator | 0.0221 | CDH2 |
| PTN | growth factor | 0.0221 | COL9A1 |
| TENM1 | transmembrane receptor | 0.0221 | CHL1 |
| WNT7A | cytokine | 0.0221 | CDH2 |
| WWC1 | transcription regulator | 0.0221 | CDH2 |
| mir-135 | microrna | 0.0221 | CDH2 |
| mir-210 | microrna | 0.0221 | CDH2 |
| mir-379 | microrna | 0.0221 | CDH2 |
| CEMIP | enzyme | 0.0246 | CDH2 |
| DEF6 | other | 0.0246 | CDH2 |
| FOXC2 | transcription regulator | 0.0246 | CDH2 |
| H3C14 | other | 0.0246 | THBS4 |
| HOXA11-AS | other | 0.0246 | CDH2 |
| POMGNT1 | enzyme | 0.0246 | CDH2 |
| RLIM | enzyme | 0.0246 | CDH2 |
| SPRY4-IT1 | other | 0.0246 | CDH2 |
| STX3 | transporter | 0.0246 | VCAN |
| TNFAIP6 | other | 0.0246 | CDH2 |
| miR-291a-3p (and other miRNAs w/seed AAGUGCU) | mature microrna | 0.0246 | C3 |
| EHMT1 | transcription regulator | 0.0256 | C3,LCN2 |
| CCAT1 | other | 0.027 | CDH2 |
| DDX17 | enzyme | 0.027 | LCN2 |
| Focal adhesion kinase | group | 0.027 | CDH2 |
| LIMA1 | other | 0.027 | VCAN |
| MGAT1 | enzyme | 0.027 | CDH2 |
| MIEF2 | other | 0.027 | CDH2 |
| PTPN6 | phosphatase | 0.027 | CDH2 |
| SNHG20 | other | 0.027 | CDH2 |
| WDR5 | transcription regulator | 0.027 | CDH2 |
| ZFAS1 | other | 0.027 | CDH2 |
| CALR | transcription regulator | 0.0294 | B4GALT1 |
| CLCA2 | ion channel | 0.0294 | CDH2 |
| DANCR | other | 0.0294 | CDH2 |
| FGF1 | growth factor | 0.0294 | PLG |
| FLCN | other | 0.0294 | TPP1 |
| NEUROD1 | transcription regulator | 0.0294 | NCAM1 |
| NR1H3 | ligand-dependent nuclear receptor | 0.0294 | APOM |
| PAK4 | kinase | 0.0294 | CDH2 |
| SOX2-OT | other | 0.0294 | CDH2 |
| miR-205-5p (and other miRNAs w/seed CCUUCAU) | mature microrna | 0.0294 | CDH2 |
| ERK | group | 0.0306 | LCN2,VCAN |
| CAPN3 | peptidase | 0.0318 | GPX3 |
| FOXD2-AS1 | other | 0.0318 | CDH2 |
| SIM2 | transcription regulator | 0.0318 | CDH2 |
| mir-322 | microrna | 0.0318 | CDH2 |
| FASN | enzyme | 0.0342 | CDH2 |
| GALNT6 | enzyme | 0.0342 | CDH2 |
| MUC4 | other | 0.0342 | CDH2 |
| PROX1 | transcription regulator | 0.0342 | SCG3 |
| SNHG11 | other | 0.0342 | CDH2 |
| USP1 | peptidase | 0.0342 | COL6A2 |
| mir-150 | microrna | 0.0342 | NCAM1 |
| CD46 | transmembrane receptor | 0.0366 | C3 |
| EZR | other | 0.0366 | CDH2 |
| NFKBIZ | transcription regulator | 0.0366 | LCN2 |
| PTP4A3 | phosphatase | 0.0366 | CDH2 |
| ST6GAL1 | enzyme | 0.0366 | CDH2 |
| TFE3 | transcription regulator | 0.0366 | TPP1 |
| mir-218 | microrna | 0.0366 | CDH2 |
| BRD7 | transcription regulator | 0.039 | VCAN |
| PTAFR | g-protein coupled receptor | 0.039 | CDH2 |
| PTTG1 | transcription regulator | 0.039 | CDH2 |
| HNF1A | transcription regulator | 0.0403 | APOH,APOM |
| BPIFB1 | other | 0.0438 | CDH2 |
| ICAM1 | transmembrane receptor | 0.0438 | CDH2 |
| PCGF2 | transcription regulator | 0.0438 | CDH2 |
| SKP2 | other | 0.0438 | CDH2 |
| POU5F1 | transcription regulator | 0.045 | CDH2,NCAM1 |
| IL6 | cytokine | 0.0461 | C3,PLG |
| HNF1A-AS1 | other | 0.0462 | CDH2 |
| HNRNPA2B1 | other | 0.0473 | NRCAM,VCAN |
| HULC | other | 0.0485 | CDH2 |
| SPZ1 | transcription regulator | 0.0485 | CDH2 |
| let-7a-5p (and other miRNAs w/seed GAGGUAG) | mature microrna | 0.0485 | CDH2 |
| mir-148 | microrna | 0.0485 | CDH2 |
